# Supplementary figures and images for: The Drosophila Duox maturation factor is a key component of a positive feedback loop that sustains regeneration signaling
Source: PLoS Genet. 2017 Jul 28;13(7):e1006937. doi: 10.1371/journal.pgen.1006937 (PMC5550008; doi:10.1371/journal.pgen.1006937)

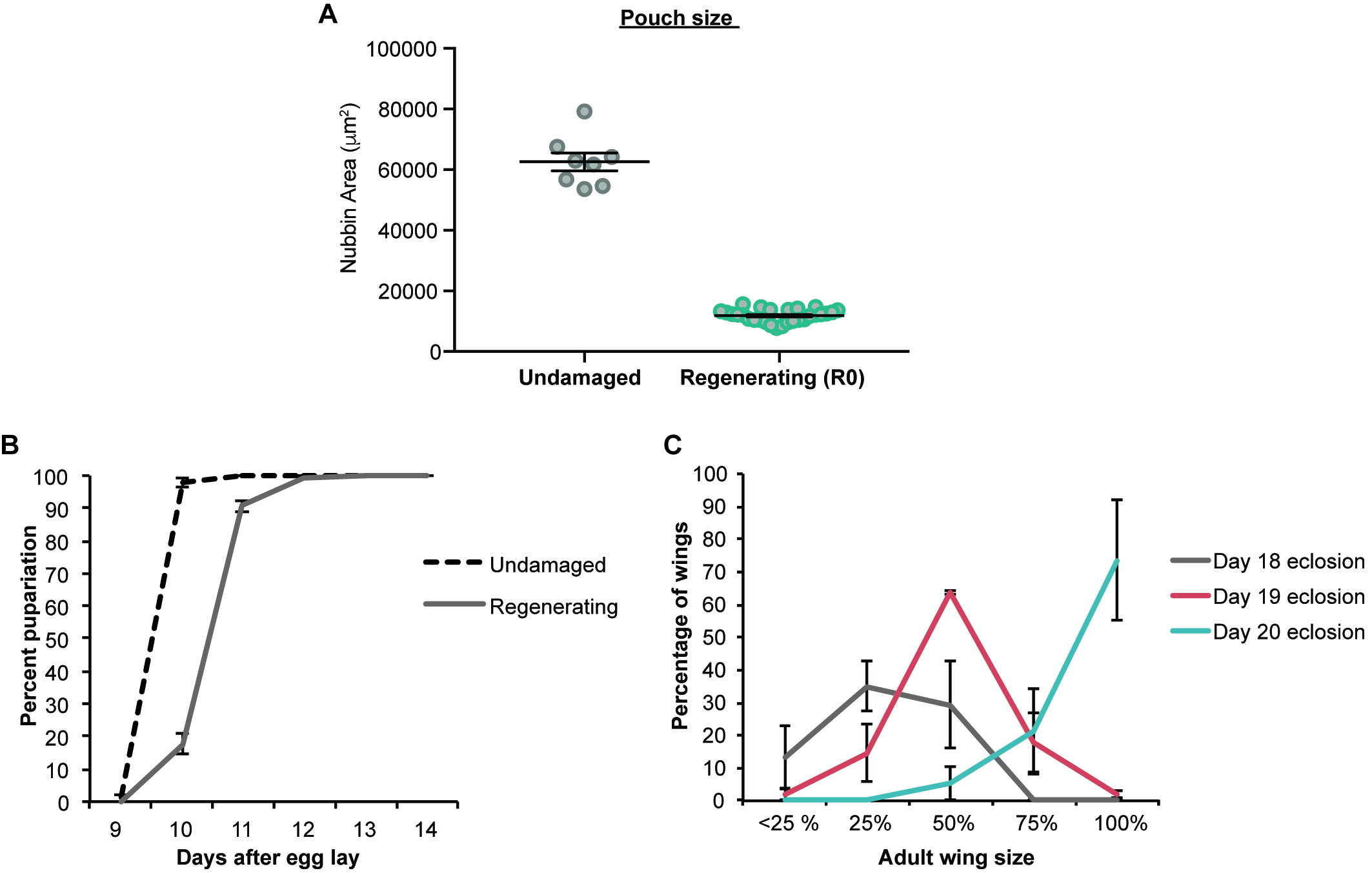

Supplement: S1 Fig — (A) Variation in the size of the wing pouch as marked by anti-Nub immunostaining in undamaged and ablated wing discs. Note the minimal variation in pouch size at R0, indicating consistency of ablation. Undamaged n = 8, regenerating n = 28. Error bars are SEM. (B) Quantification of percentage of regenerating animals that had pupariated by each day, showing the asynchronous progression to pupariation that occurred after tissue damage. Undamaged animals were w1118;; rnGAL4, Gal80TS/+, regenerating animals were w1118;; rnGAL4, UAS-reaper, Gal80TS/+, and both experienced the 24-hour temperature shift. Three independent experiments, total undamaged n = 144 pupae, regenerating n = 176 pupae. (C) Percentage of wings of different sizes on w1118;; rnGAL4, UAS-reaper, Gal80TS/+ animals that eclosed on different days after egg laying due the asynchronous development induced by tissue damage. Note that the animals that eclosed first (day 18) had smaller wings than those that eclosed on day 19, and those that eclosed on day 20 had the largest wings. Thus, variation in wing size after disc regeneration is partly determined by length of time for regeneration. Three independent experiments, total n = 371 wings. (TIF) [file pgen.1006937.s001.tif]

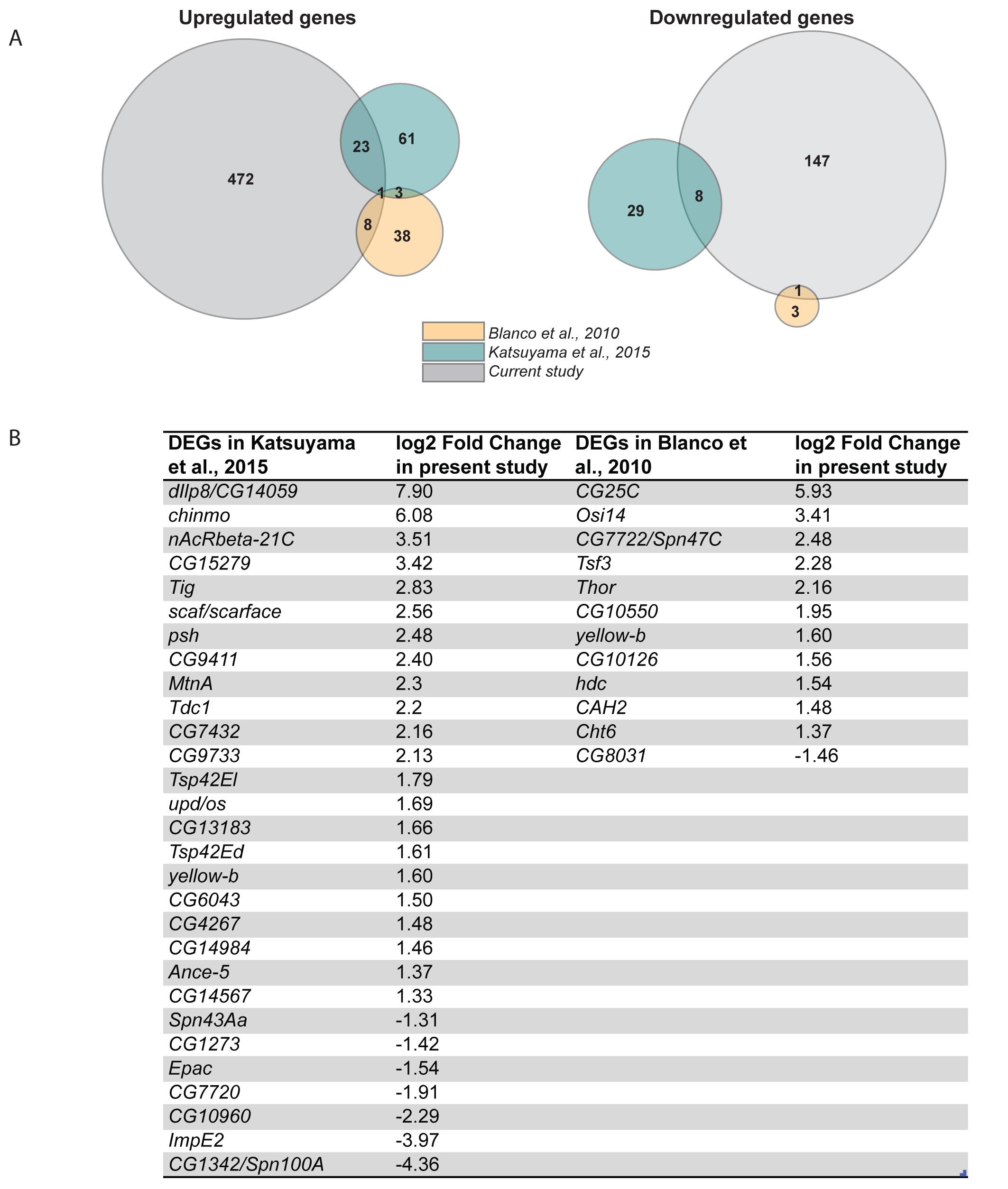

Supplement: S2 Fig — (A) Venn diagrams showing genes at least 1.3-fold upregulated or downregulated in three transcription profiles (this work, [39,40]) of regenerating wing discs generated using different methods. Note that the number of genes from the Blanco et al. study is an underrepresentation because the complete list of differentially expressed genes identified was not published. (B) Lists of differentially expressed genes (DEGs) in common between this study and each prior study, along with fold change in this study. (TIF) [file pgen.1006937.s002.tif]

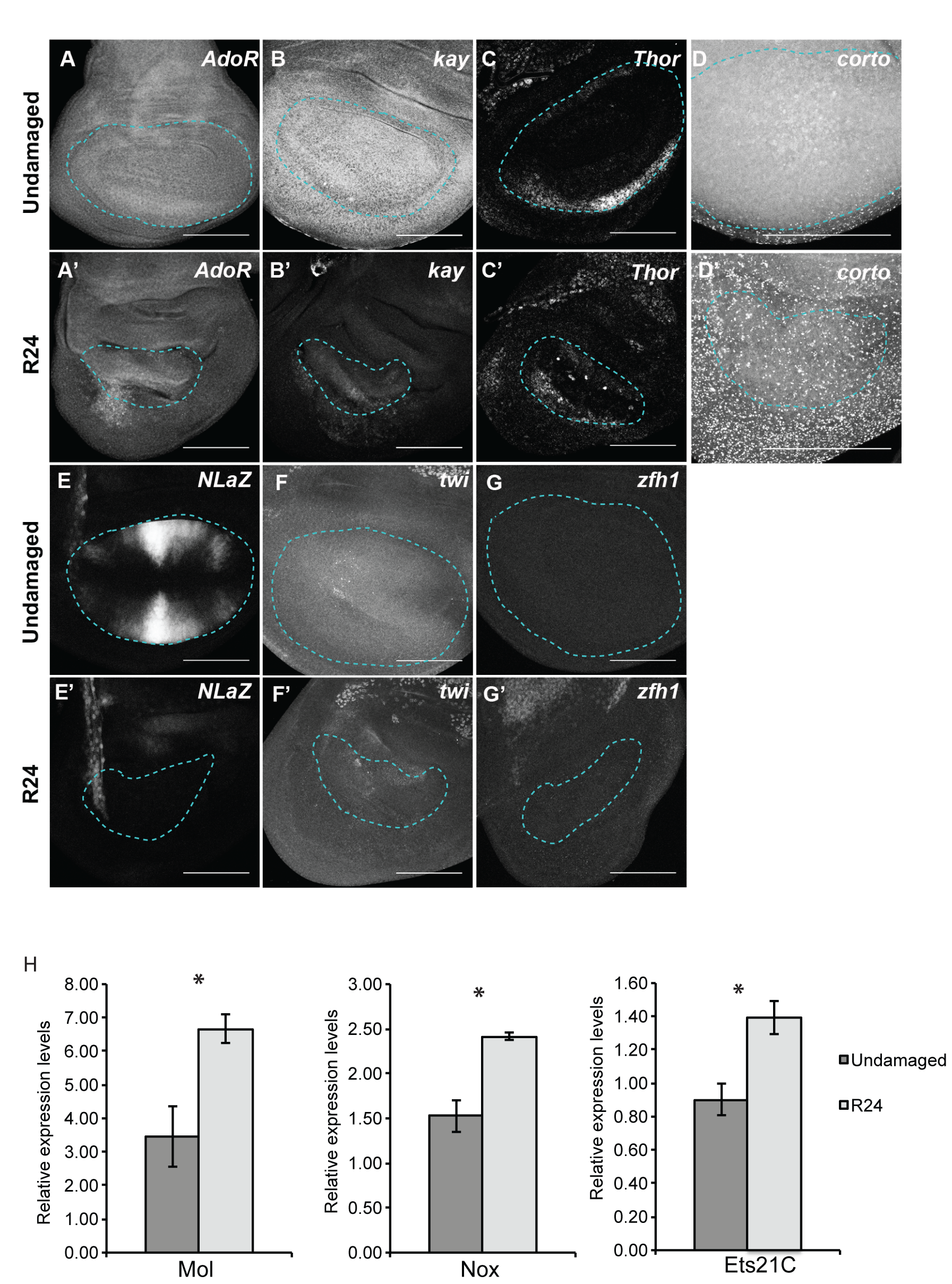

Supplement: S3 Fig — Undamaged (A-G) and regenerating (R24) (A’-G’) wing discs. (A-A’) AdoR-GFP MiMIC enhancer trap. (B-B’) Kayak-GFP protein trap. (C-C’) Thor-lacZ enhancer trap. (D-D’) Corto-GFP protein trap. (E-E’) NLaz-GFP MiMIC enhancer trap. (F-F’) anti-Twist. (G-G’) zfh1-lacZ enhancer trap. Blue dashed line outlines the wing primordium. Scale bars are 100 μm. (H) Quantification of upregulation of mol, Nox, and Ets21C expression using qPCR. Four biological replicates each. Error bars are SEM, *p<0.05. (TIF) [file pgen.1006937.s003.tif]

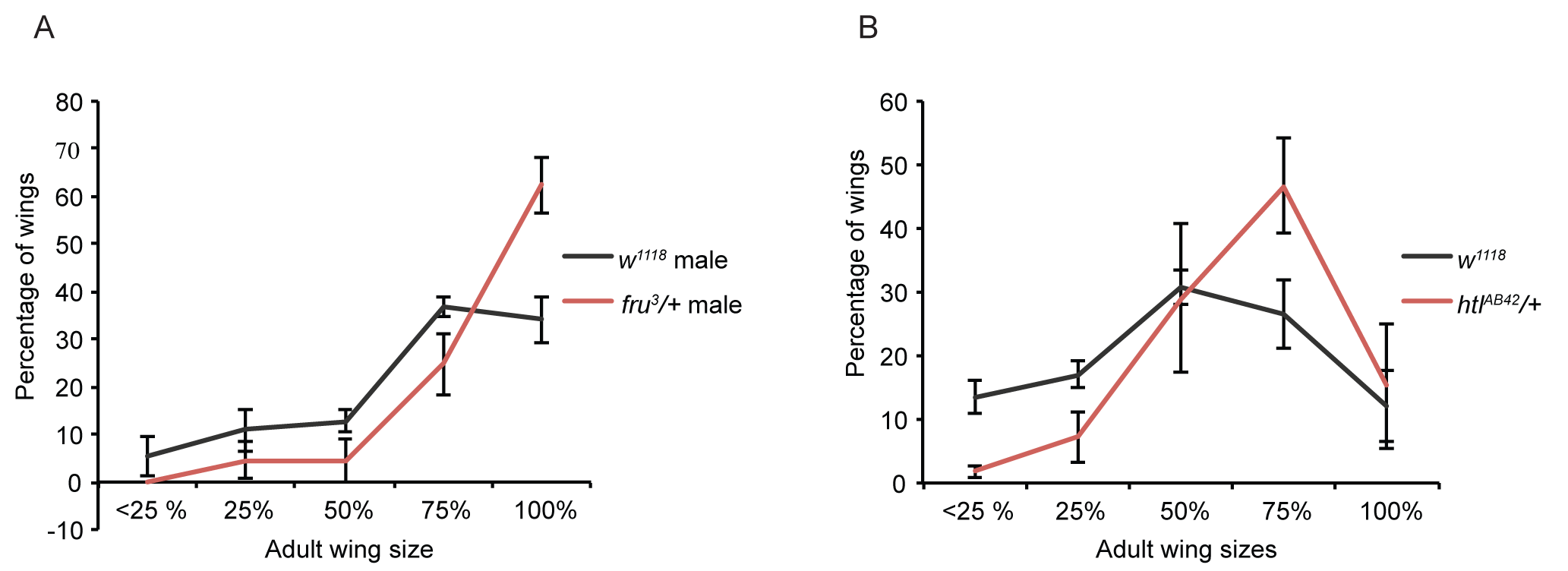

Supplement: S4 Fig — (A) Adult fru3/+ males had larger wings after regeneration than controls. Three independent experiments, w1118 n = 112 wings, fru3/+ n = 95 wings, p<0.001 by a chi-squared test. (B) Adult htlAB42/+ animals had larger wings after regeneration than controls. Three independent experiments, w1118 n = 316 wings, htlAB42/+ n = 223 wings, p<0.001 by a chi-squared test. Error bars are SEM. (TIF) [file pgen.1006937.s004.tif]

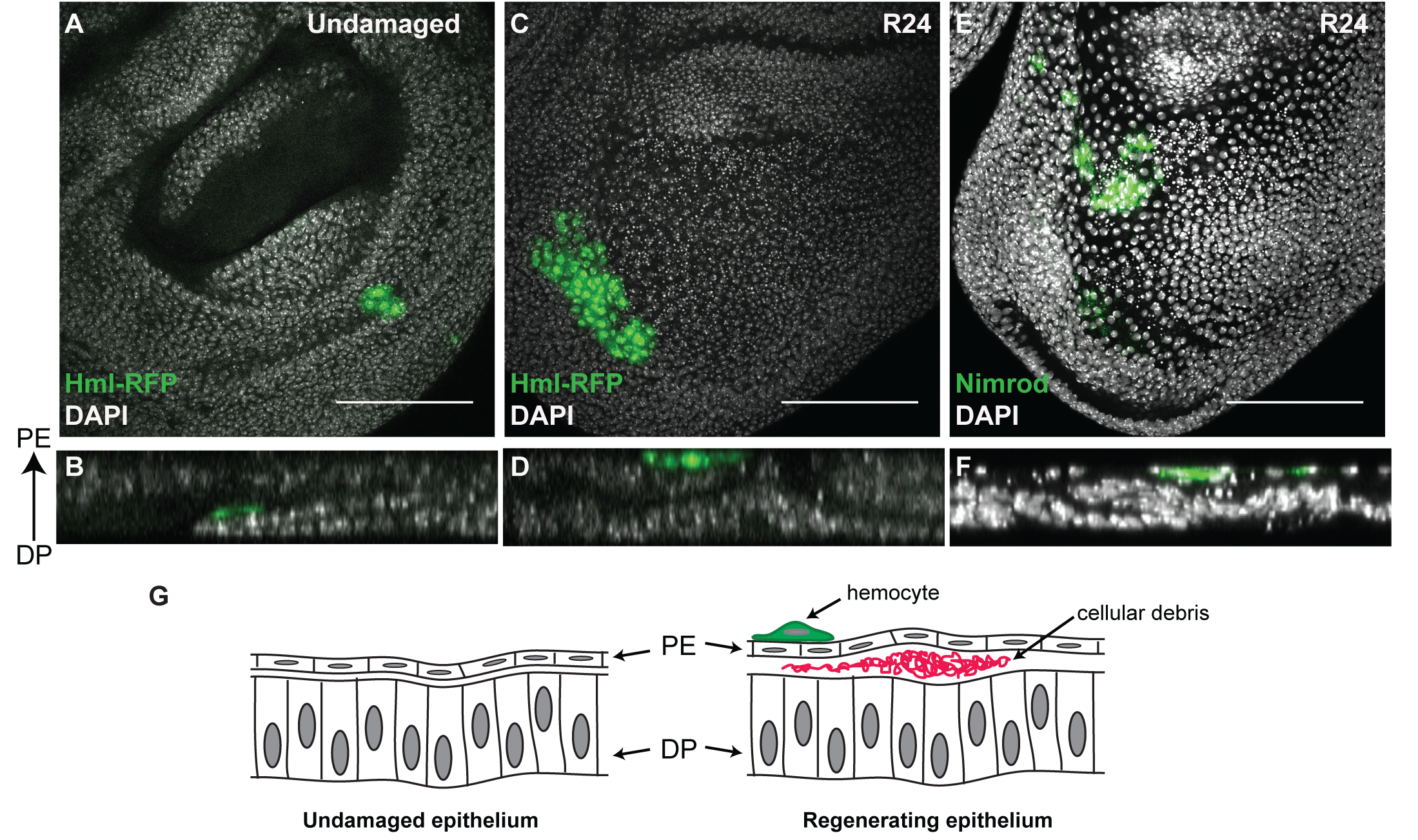

Supplement: S5 Fig — (A-D) Hemolectin-RFP (Hml-RFP) (green) showing hemocytes near undamaged (A,B) and R24 (C,D) wing discs. (E,F) Anti-Nimrod (green) also showing hemocytes near an R24 wing disc, confirming the Hml-RFP results. (B), (D), and (F) are orthogonal slices with the columnar epithelium or the disc proper (DP) toward the bottom and the peripodial epithelium (PE) toward the top of the images. (G) Schematic of an undamaged and a regenerating epithelium showing the location of a hemocyte outside the PE. (TIF) [file pgen.1006937.s005.tif]

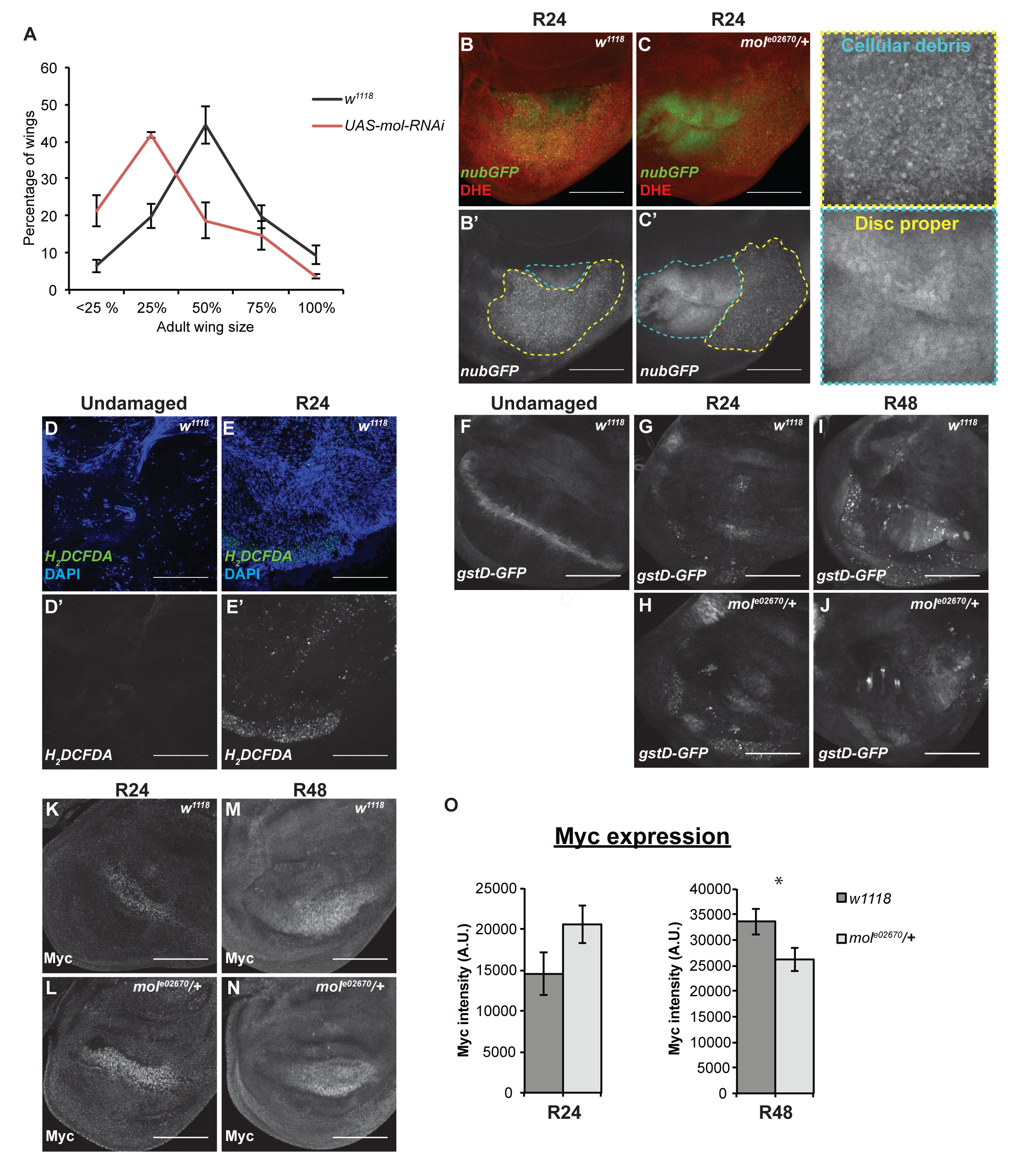

Supplement: S6 Fig — (A) Expression of molRNAi caused smaller adult wings after regeneration of the imaginal discs than controls. Three independent experiments, w1118 n = 402 wings, UAS-molRNAi n = 221 wings, p<0.0001 by a chi-squared test. (B-C) Cellular debris is visually distinct from the regenerating epithelium. Regenerating w1118 (B) and mole02670/+ (C) discs at R24, expressing the nub-GFP enhancer trap and stained with DHE. The speckled, grainy GFP tissue is cellular debris, outlined with yellow. The smooth GFP tissue is the intact epithelium, outlined with blue. The side panels are zoomed-in views of debris and epithelium, to show that they are easily distinguished. (D,E) Undamaged and R24 w1118 discs stained with DAPI and the ROS detector H2DCFDA. (F-J) gstD-GFP expression in undamaged w1118 (F), and regenerating w1118 (G,H) and mole02670/+ (I,J) wing discs at R24 (G,I) and R48 (H,J). (K-O) Anti-Myc immunostaining in w1118 (K,L) and mole02670/+ (M,N) regenerating wing discs at R24 (K,M) and R48 (L,N). (O) Quantification of immunofluorescence in Myc staining. R24 w1118 n = 14 discs, mole02670/+ n = 12 discs. R48 w1118 n = 11 discs, mole02670/+ n = 14 discs. Scale bars are 100 μm. Error bars are SEM except where noted. *p<0.02. (TIF) [file pgen.1006937.s006.tif]

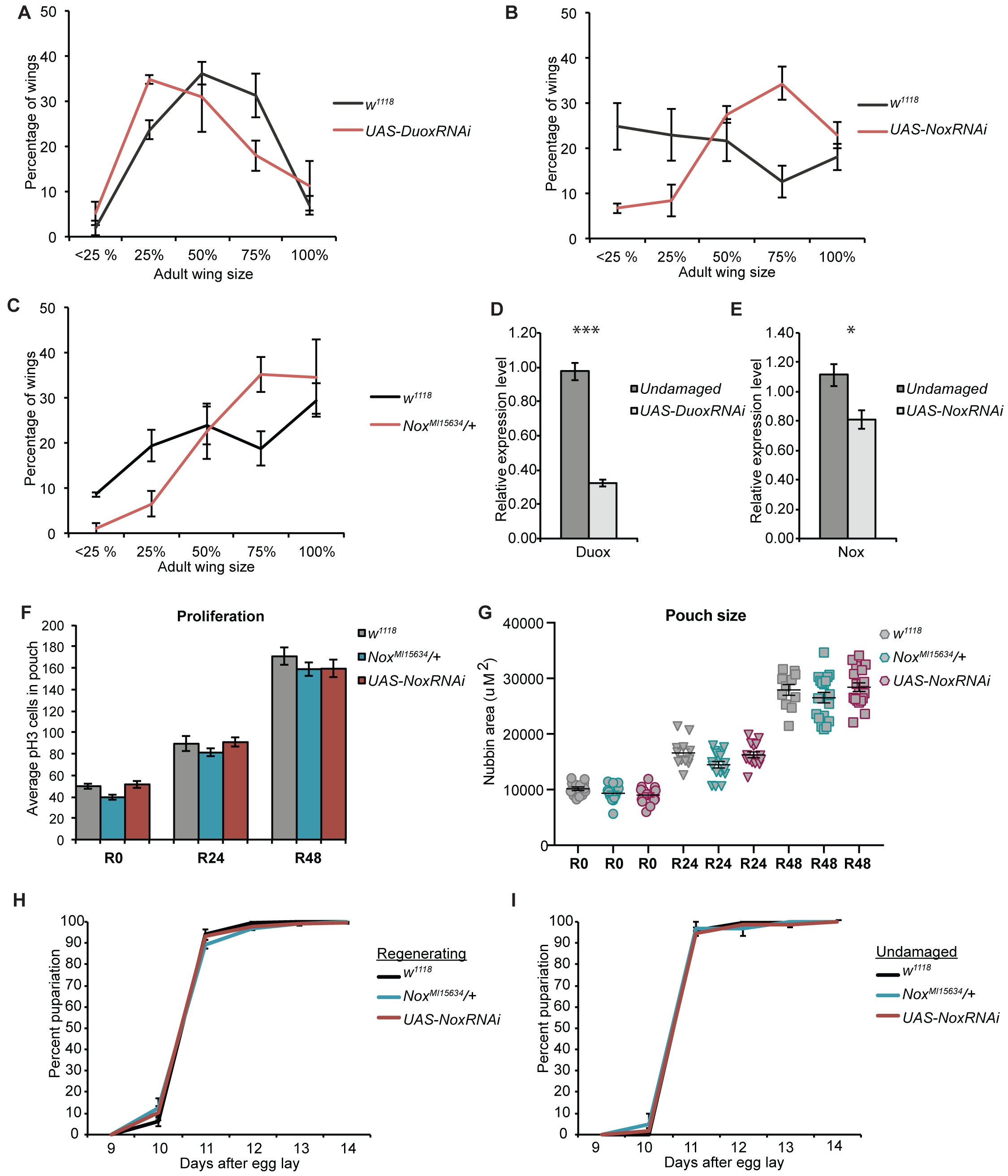

Supplement: S7 Fig — Regeneration assays using adult wing size to assess extent of regenerative growth in the imaginal discs. (A) Sizes of adult wings after disc regeneration in w1118 and UAS-DuoxRNAi animals. Three independent experiments. w1118 n = 390 wings, UAS-DuoxRNAi n = 200 wings, p = 0.0005 using a chi-squared test. (B) Sizes of adult wings after disc regeneration in w1118 and UAS-NoxRNAi animals. Two independent experiments, thus error bars are SD. w1118 n = 299 wings, UAS-NoxRNAi n = 257 wings, p<0.0001 using a chi-squared test. (C) Sizes of adult wings after disc regeneration in w1118 and NoxMI15634/+ animals. Three independent experiments. w1118 n = 349 wings, NoxMI15634/+ n = 180 wings, p<0.0001 using a chi-squared test. (D,E) qPCR showing effectiveness of Duox (D) and Nox (E) RNAi. The RNAi was expressed under rn-GAL4 control in the pouch of normally developing wing discs for 24 hours before collecting for qPCR. Three biological replicates each, *p<0.05. (F) Total number of mitotic cells as identified by anti-phospho-Histone H3 staining in the wing pouch as identified by anti-Nub staining in the indicated genotypes. R0 w1118 n = 14 discs, NoxMI15634/+ n = 15 discs, UAS-NoxRNAi n = 15 discs, R24 w1118 n = 12 discs, NoxMI15634/+ n = 17 discs, UAS-NoxRNAi n = 15 discs, R48 w1118 n = 11 discs, NoxMI15634/+ n = 18 discs, UAS-NoxRNAi n = 19 discs. (G) Area of the wing pouch as marked by anti-Nub staining was measured at R0, R24 and R48 for the indicated genotypes. R0 w1118 n = 14 discs, NoxMI15634/+ n = 15 discs, UAS-NoxRNAi n = 15 discs, R24 w1118 n = 12 discs, NoxMI15634/+ n = 17 discs, UAS-NoxRNAi n = 15 discs, R48 w1118 n = 11 discs, NoxMI15634/+ n = 18 discs, UAS-NoxRNAi n = 19 discs. (H) Pupariation timing for regenerating animals of the indicated gentoypes. Three independent experiments. w1118 n = 204, NoxMI15634/+ n = 113, UAS-NoxRNAi n = 206 (I) Pupariation timing for normally developing animals that did not experience the thermal shift and so did not ablate and regene [file pgen.1006937.s007.tif]

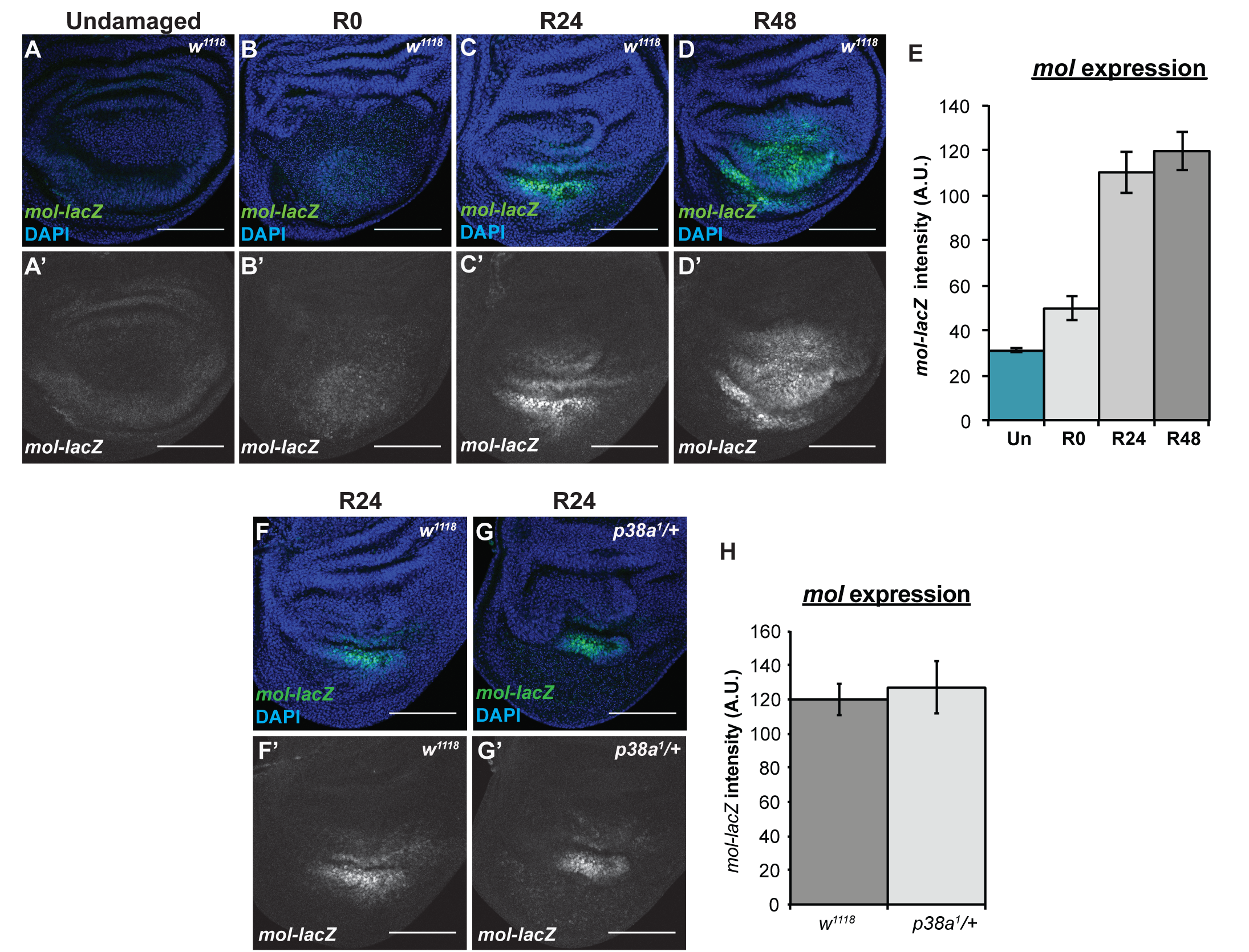

Supplement: S8 Fig — (A-E) Anti-β-galacosidase immunostaining showing expression of the mol-lacZ reporter (green) in w1118 undamaged discs (A), and in regenerating wing discs at R0 (B), R24 (C) and R48 (D). (E) Quantification of mol-lacZ expression changes. Undamaged n = 3, R0 n = 7, R24 n = 10, R48 n = 10. (F,G) Anti-β-galacosidase immunostaining showing expression of the mol-lacZ reporter (green) in w1118 (F) and p38a1/+ (G) R24 discs. (H) Quantification of the fluorescence from the immunostaining. Two independent experiments, for a total w1118 n = 10 discs, p38a1/+ n = 12 discs. Scale bars are 100 μm. Error bars are SEM. (TIF) [file pgen.1006937.s008.tif]
