# Supplementary material for: The Drosophila Duox maturation factor is a key component of a positive feedback loop that sustains regeneration signaling
Source: PLoS Genet. 2017 Jul 28;13(7):e1006937. doi: 10.1371/journal.pgen.1006937 (PMC5550008; doi:10.1371/journal.pgen.1006937)
Supplement: S1 Table — (PDF) [file pgen.1006937.s009.pdf]

Table S1: List of Upregulated genes (log2 fold change ≥ 1.3, p<0.05)

#	gene_id	gene	locus	log2(fold_change)	p_value	q_value
1	CG10513	CG10513	3R:21101510-21102934	inf	5.0000E-05	2.7364E-04
2	CG12655	CG12655	X:19852421-19853421	inf	5.0000E-05	2.7364E-04
3	CG13067	CG13067	3L:16264401-16265018	inf	5.0000E-05	2.7364E-04
4	CG32603	CG32603	X:14078573-14079840	inf	5.0000E-05	2.7364E-04
5	CG34330	CG34330	X:18962305-18962925	inf	5.0000E-05	2.7364E-04
6	CG5999	CG5999	3R:8567679-8569402	inf	5.0000E-05	2.7364E-04
7	CG8193	CG8193	2R:4929764-4932213	inf	5.0000E-05	2.7364E-04
8	MtnC	MtnC	3R:16186029-16186356	inf	5.0000E-05	2.7364E-04
9	CG14059	CG14059	3L:17020620-17025180	7.92	5.0000E-05	2.7364E-04
10	chinmo	chinmo	2L:1651259-1698617	6.08	5.0000E-05	2.7364E-04
11	Cg25C	Cg25C	2L:5029614-5037114	5.93	5.0000E-05	2.7364E-04
12	vkg	vkg	2L:5012143-5027412	5.46	5.0000E-05	2.7364E-04
13	Lim1	Lim1	X:8651267-8699808	5.21	2.6000E-03	8.7988E-03
14	CG9572	CG9572	X:20042158-20049547	4.93	5.5000E-04	2.3101E-03
15	htl	htl	3R:13870935-13878706	4.59	5.0000E-05	2.7364E-04
16	CG9259	CG9259	2L:21089564-21090999	4.55	4.1000E-03	1.2940E-02
17	CG9336	CG9336	2L:20859168-20861017	4.49	5.0000E-05	2.7364E-04
18	CG9650	CG9650	X:7087108-7134453	4.39	5.5000E-04	2.3101E-03
19	CG3690	CG3690	X:843322-845915	4.26	2.6000E-03	8.7988E-03
20	alphaTub85E	alphaTub85E	3R:5556937-5558970	4.24	5.0000E-05	2.7364E-04
21	twi	twi	2R:18933630-18935849	4.23	5.0000E-05	2.7364E-04
22	CG6142	CG6142	3R:21958875-21962870	4.15	5.0000E-05	2.7364E-04
23	Cpr76Bc	Cpr76Bc	3L:19514366-19520374	4.09	5.7000E-03	1.7192E-02
24	pinta	pinta	3R:18249026-18250447	4.05	5.0000E-05	2.7364E-04
25	CG3973	CG3973	X:6494097-6546131	3.97	5.0000E-05	2.7364E-04
26	Cyp18a1	Cyp18a1	X:18582569-18586778	3.82	5.0000E-05	2.7364E-04
27	Aph-4	Aph-4	3R:26874653-26878268	3.82	5.0000E-05	2.7364E-04
28	CG13041	CG13041	3L:16312053-16312621	3.80	1.5500E-03	5.6919E-03
29	Ets21C	Ets21C	2L:547413-552454	3.78	5.0000E-05	2.7364E-04
30	Aldh	Aldh	2L:9387388-9391679	3.78	5.0000E-05	2.7364E-04
31	Him	Him	X:18103463-18104774	3.75	5.0000E-05	2.7364E-04
32	ftz-f1	ftz-f1	3L:18745013-18791831	3.75	5.0000E-05	2.7364E-04
33	CG1208	CG1208	3R:1655783-1664504	3.73	5.0000E-05	2.7364E-04
34	AP-2	AP-2	3L:21591957-21606453	3.63	5.0000E-05	2.7364E-04
35	CG30046	CG30046	2R:8201623-8204736	3.60	4.5000E-04	1.9354E-03
36	zfh1	zfh1	3R:26591647-26612899	3.51	5.0000E-05	2.7364E-04
37	nAcRbeta-21C	nAcRbeta-21C	2L:545147-547096	3.51	5.0000E-05	2.7364E-04
38	CG6579	CG6579	2L:12175447-12177886	3.49	5.0000E-05	2.7364E-04

39	mol	mol	2L:14975746-14997556	3.48	5.0000E-05	2.7364E-04
40	CG4998	CG4998	3L:16332098-16338348	3.44	5.0000E-05	2.7364E-04
41	CG15279	CG15279	2L:14844992-14851749	3.42	5.0000E-05	2.7364E-04
42	CG9737	CG9737	3R:26066984-26069032	3.41	1.0000E-04	5.1606E-04
43	Osi14	Osi14	3R:2124853-2126472	3.41	5.0000E-05	2.7364E-04
44	SK	SK	X:5234270-5295156	3.39	5.0000E-05	2.7364E-04
45	Osi15	Osi15	3R:2127527-2129375	3.33	5.0000E-05	2.7364E-04
46	fru	fru	3R:14242314-14371308	3.32	5.0000E-05	2.7364E-04
47	stumps	stumps	3R:10402940-10433804	3.32	5.0000E-05	2.7364E-04
48	CG7365	CG7365	3L:20117395-20120465	3.28	5.0000E-05	2.7364E-04
49	CG9338	CG9338	2L:20865226-20867311	3.25	5.0000E-05	2.7364E-04
50	Chit	Chit	2R:12560965-12618889	3.25	5.9000E-03	1.7692E-02
51	CG13631	CG13631	3R:20595075-20595833	3.24	4.5500E-03	1.4187E-02
52	Cpr49Ah	Cpr49Ah	2R:8301466-8302675	3.23	5.0000E-05	2.7364E-04
53	Ir87a	Ir87a	3R:9245994-9248862	3.20	5.0000E-05	2.7364E-04
54	CG11951	CG11951	3R:25061085-25064394	3.19	5.0000E-05	2.7364E-04
55	CG13611	CG13611	3R:20105293-20107299	3.16	5.0000E-05	2.7364E-04
56	A3-3	A3-3	X:1136772-1168682	3.15	5.0000E-05	2.7364E-04
57	Cpr64Aa	Cpr64Aa	3L:4207837-4209178	3.12	5.0000E-05	2.7364E-04
58	Oseg5	Oseg5	2L:20831261-20835607	3.09	7.1000E-03	2.0705E-02
59	CG30359	CG30359	2R:4350277-4352597	3.08	2.0000E-04	9.4764E-04
60	CG18585	CG18585	2L:7647052-7691057	3.01	5.2500E-03	1.5995E-02
61	CG9932	CG9932	2L:13022670-13060115	2.97	5.0000E-05	2.7364E-04
62	CG1368	CG1368	X:14068793-14069703	2.96	5.0000E-05	2.7364E-04
63	CG17739	CG17739	2R:8194028-8197917	2.93	5.0000E-05	2.7364E-04
64	yellow-e	yellow-e	3R:9235263-9241006	2.92	5.0000E-05	2.7364E-04
65	Cda9	Cda9	2R:13016337-13017839	2.91	6.6500E-03	1.9589E-02
66	fu12	fu12	2L:8449690-8463990	2.90	5.0000E-05	2.7364E-04
67	CG11670	CG11670	3R:8864134-8870123	2.89	1.0000E-02	2.7615E-02
68	CG31103	CG31103	3R:21045068-21047095	2.88	5.0000E-05	2.7364E-04
69	CG15155	CG15155	2L:18154863-18155942	2.85	1.5000E-04	7.3812E-04
70	Mes2	Mes2	3L:22938758-22945346	2.85	2.0000E-04	9.4764E-04
71	CG30039	CG30039	2R:7938479-7939260	2.83	6.7500E-03	1.9833E-02
72	Tig	Tig	2L:6412314-6423308	2.83	5.0000E-05	2.7364E-04
73	CG15213	CG15213	3L:5125895-5126320	2.82	1.0900E-02	2.9783E-02
74	CG6652	CG6652	3L:17013439-17015956	2.81	5.0000E-05	2.7364E-04
75	CG8927	CG8927	3R:12006741-12012615	2.80	1.5000E-04	7.3812E-04
76	CG18417	CG18417	3L:7393176-7394774	2.77	1.3500E-03	5.0775E-03
77	CG32379	CG32379	3L:7389454-7391309	2.76	7.9000E-03	2.2620E-02
78	CG17738	CG17738	3R:8084470-8128509	2.75	2.9500E-03	9.8088E-03
79	Mhc	Mhc	2L:16766736-16788766	2.75	5.0000E-05	2.7364E-04
80	CG31556	CG31556	3R:2132966-2135018	2.75	2.0000E-04	9.4764E-04
81	dei	dei	3R:22267048-22274716	2.74	3.0000E-04	1.3573E-03

82	CG33205	CG33205	3L:9809169-9827447	2.74	5.0000E-05	2.7364E-04
83	CG14968	CG14968	3L:3299335-3303108	2.74	5.0000E-05	2.7364E-04
84	CG6356	CG6356	3R:20109855-20115540	2.69	5.0000E-05	2.7364E-04
85	CG13321	CG13321	2R:8841060-8842675	2.69	7.2000E-03	2.0963E-02
86	CG9222	CG9222	2L:6131022-6132484	2.68	4.0000E-04	1.7372E-03
87	CG13044	CG13044	3L:16295590-16296260	2.67	5.0000E-05	2.7364E-04
88	CG15080	CG15080	2R:14670805-14682105	2.64	5.0000E-05	2.7364E-04
89	CG13640	CG13640	3R:20687380-20687884	2.64	9.7500E-03	2.7013E-02
90	CG16820	CG16820	2L:13246230-13250193	2.63	5.0000E-05	2.7364E-04
91	CG13157	CG13157	2R:8303959-8305647	2.63	5.0000E-05	2.7364E-04
92	CG8916	CG8916	X:15815987-15818591	2.61	9.5000E-04	3.7311E-03
93	CG14304	CG14304	3R:14418739-14447049	2.58	5.0000E-05	2.7364E-04
94	Obp99a	Obp99a	3R:25497381-25498056	2.58	5.0000E-05	2.7364E-04
95	spz5	spz5	3L:2883637-2892164	2.57	1.0000E-03	3.9012E-03
96	scarface	scarface	2R:1564253-1579200	2.56	5.0000E-05	2.7364E-04
97	Cda5	Cda5	2L:25401-59242	2.56	5.0000E-05	2.7364E-04
98	zormin	zormin	3L:2117465-2150089	2.52	5.0000E-05	2.7364E-04
99	CG3640	CG3640	2R:20527210-20529760	2.51	1.3500E-02	3.5442E-02
100	CG6045	CG6045	3R:11368473-11373457	2.51	5.0000E-05	2.7364E-04
101	CG13954	CG13954	2R:5257634-5261741	2.50	1.0000E-04	5.1606E-04
102	CG31030	CG31030	3R:25990413-25992152	2.48	5.0000E-05	2.7364E-04
103	Ect3	Ect3	3R:7819896-7831194	2.48	5.0000E-05	2.7364E-04
104	CG18410	CG18410	3R:20617464-20625390	2.48	5.0000E-05	2.7364E-04
105	CG6788	CG6788	X:17782209-17783437	2.48	6.5000E-04	2.6711E-03
106	CG7722	CG7722	2R:6827692-6829206	2.48	5.0000E-05	2.7364E-04
107	psh	psh	X:18378514-18380954	2.48	5.0000E-05	2.7364E-04
108	CG30471	CG30471	2R:11422699-11425155	2.46	2.1500E-03	7.5077E-03
109	CG14572	CG14572	3L:21734638-21735541	2.44	1.3750E-02	3.6017E-02
110	TwdlM	TwdlM	3R:22444844-22445953	2.44	1.6000E-03	5.8616E-03
111	CG11353	CG11353	3L:4533171-4539281	2.43	5.0000E-05	2.7364E-04
112	Cpr51A	Cpr51A	2R:10400046-10402713	2.42	5.0000E-05	2.7364E-04
113	lbl	lbl	3R:17235700-17259845	2.42	6.0000E-04	2.4964E-03
114	CG7532	CG7532	2L:14349113-14351510	2.41	5.0000E-05	2.7364E-04
115	CG11370	CG11370	3L:22705256-22706763	2.40	5.0000E-05	2.7364E-04
116	CG9411	CG9411	X:14366435-14374112	2.40	5.0000E-05	2.7364E-04
117	CG34283	CG34283	3R:14531173-14558068	2.39	1.4550E-02	3.7721E-02
118	Mur18B	Mur18B	X:19107288-19108971	2.38	3.1000E-03	1.0216E-02
119	CG2837	CG2837	2L:4904067-4906761	2.38	5.0000E-05	2.7364E-04
120	LamC	LamC	2R:10414075-10472826	2.37	5.0000E-05	2.7364E-04
121	TwdlBeta	TwdlBeta	2R:7750041-7750753	2.37	1.5000E-04	7.3812E-04
122	CG30203	CG30203	2R:8198193-8201340	2.37	5.0000E-05	2.7364E-04
123	CG10175	CG10175	3R:19348422-19356704	2.35	5.0000E-05	2.7364E-04
124	Cht7	Cht7	3L:3090984-3106956	2.35	5.0000E-05	2.7364E-04

125	CG31427	CG31427	3R:25064551-25066085	2.35	5.0000E-05	2.7364E-04
126	CG6108	CG6108	2L:13261009-13286409	2.35	1.1500E-03	4.4143E-03
127	CG34333	CG34333	X:2170005-2171743	2.35	1.5000E-04	7.3812E-04
128	Obp50b	Obp50b	2R:10258690-10260511	2.32	6.1500E-03	1.8336E-02
129	Ubx	Ubx	3R:12482097-12560348	2.30	5.0000E-05	2.7364E-04
130	MtnA	MtnA	3R:5609486-5610093	2.30	5.0000E-05	2.7364E-04
131	fs(1)Ya	fs(1)Ya	X:2618318-2620614	2.30	5.0000E-04	2.1172E-03
132	CG17234	CG17234	2L:2253078-2253834	2.30	1.3900E-02	3.6329E-02
133	Sp212	Sp212	3R:10047795-10049877	2.29	2.0000E-04	9.4764E-04
134	CG3349	CG3349	3L:14973208-14975443	2.29	5.0000E-05	2.7364E-04
135	Tsf3	Tsf3	2R:12110718-12121591	2.28	1.0000E-04	5.1606E-04
136	CG15353	CG15353	2L:2006762-2007193	2.27	5.0000E-05	2.7364E-04
137	CG17752	CG17752	3R:15435946-15438159	2.26	1.7000E-03	6.1650E-03
138	dnr1	dnr1	2R:18451011-18480473	2.25	5.0000E-05	2.7364E-04
139	CG4301	CG4301	X:16303110-16308431	2.25	5.0000E-05	2.7364E-04
140	Corin	Corin	2R:3433166-3448759	2.25	5.0000E-05	2.7364E-04
141	l(2)03659	l(2)03659	2R:5277962-5282705	2.24	5.0000E-05	2.7364E-04
142	Tdc1	Tdc1	2R:2561113-2580099	2.24	8.4000E-03	2.3760E-02
143	mid	mid	2L:5461640-5467609	2.24	5.0000E-05	2.7364E-04
144	Cyp28d1	Cyp28d1	2L:5210460-5214730	2.23	7.0000E-04	2.8502E-03
145	CAP	CAP	2R:6154216-6194529	2.22	5.0000E-05	2.7364E-04
146	CG7715	CG7715	3R:14531173-14558068	2.22	1.0000E-02	2.7615E-02
147	CG7025	CG7025	2L:7691108-7692683	2.21	2.3500E-03	8.0730E-03
148	CG30148	CG30148	2R:16434534-16435186	2.21	3.9500E-03	1.2526E-02
149	Obp18a	Obp18a	X:19029112-19029901	2.21	1.0000E-04	5.1606E-04
150	CG13054	CG13054	3L:16248939-16250291	2.20	3.3500E-03	1.0890E-02
151	apt	apt	2R:19452419-19488310	2.20	5.0000E-05	2.7364E-04
152	Sr-CI	Sr-CI	2L:4121720-4124002	2.20	5.0000E-05	2.7364E-04
153	phm	phm	X:18579801-18582257	2.20	5.0000E-05	2.7364E-04
154	CG10211	CG10211	2L:18517492-18531782	2.19	5.0000E-05	2.7364E-04
155	CG12068	CG12068	3R:25620357-25625055	2.19	3.0000E-04	1.3573E-03
156	CG33301	CG33301	2L:10049481-10050956	2.18	5.0000E-05	2.7364E-04
157	CG3244	CG3244	2L:4796583-4801923	2.18	5.0000E-05	2.7364E-04
158	Ir21a	Ir21a	2L:21918-25151	2.18	5.0000E-05	2.7364E-04
159	CG15212	CG15212	3L:5124649-5125227	2.17	5.0000E-05	2.7364E-04
160	RluA-1	RluA-1	2L:10463335-10474347	2.17	1.0000E-04	5.1606E-04
161	Tsp42Eg	Tsp42Eg	2R:2916417-2920741	2.17	1.0000E-04	5.1606E-04
162	Thor	Thor	2L:3478433-3479612	2.16	5.0000E-05	2.7364E-04
163	CG7432	CG7432	3R:15490817-15496549	2.16	5.0000E-05	2.7364E-04
164	CG31099	CG31099	3R:21135150-21136665	2.16	1.0000E-03	3.9012E-03
165	CG3091	CG3091	X:2146364-2148163	2.16	4.5000E-04	1.9354E-03
166	Gs2	Gs2	X:11316887-11324228	2.15	5.0000E-05	2.7364E-04
167	CG16727	CG16727	3R:15439102-15441506	2.14	5.0000E-05	2.7364E-04

168	CG31198	CG31198	3R:17580045-17583490	2.14	5.0000E-05	2.7364E-04
169	CG13833	CG13833	3R:18859005-18861285	2.13	1.0000E-04	5.1606E-04
170	CG31817	CG31817	2L:16330622-16337807	2.13	5.0000E-05	2.7364E-04
171	CG9733	CG9733	3R:26069697-26072425	2.13	5.0000E-05	2.7364E-04
172	BM-40-SPARC	BM-40-SPARC	3R:22694953-22697738	2.12	5.0000E-05	2.7364E-04
173	Tsp42Eh	Tsp42Eh	2R:2921067-2922424	2.12	1.8500E-03	6.6214E-03
174	CG9698	CG9698	3R:26340545-26342641	2.11	1.1500E-03	4.4143E-03
175	Hsromega	Hsromega	3R:17122344-17136428	2.10	5.0000E-05	2.7364E-04
176	orb	orb	3R:19090363-19106571	2.10	1.0000E-04	5.1606E-04
177	CG14879	CG14879	3R:12171854-12174946	2.09	5.0000E-05	2.7364E-04
178	CG13278	CG13278	2L:16758349-16760138	2.09	7.0000E-04	2.8502E-03
179	ste24c	ste24c	2R:12861464-12886099	2.09	7.0000E-04	2.8502E-03
180	CG13035	CG13035	3L:16389661-16393157	2.09	5.0000E-05	2.7364E-04
181	beat-IIIc	beat-IIIc	2L:17189809-17260732	2.09	3.0000E-04	1.3573E-03
182	CG1136	CG1136	3L:4046610-4059665	2.06	9.0000E-04	3.5694E-03
183	CG31445	CG31445	3R:25066301-25070220	2.06	5.0000E-05	2.7364E-04
184	CG15531	CG15531	3R:26019926-26021950	2.06	1.0000E-04	5.1606E-04
185	CG7458	CG7458	3L:21948210-21951141	2.06	6.0000E-04	2.4964E-03
186	CG31924	CG31924	2L:1352969-1355924	2.06	6.5000E-03	1.9213E-02
187	Osi2	Osi2	3R:2036720-2039214	2.05	5.0000E-05	2.7364E-04
188	CG14946	CG14946	2L:12063522-12066772	2.05	5.0000E-05	2.7364E-04
189	Obp57e	Obp57e	2R:16435320-16435796	2.05	2.8500E-03	9.5307E-03
190	CG32082	CG32082	3L:11129148-11156742	2.04	5.0000E-05	2.7364E-04
191	Ntl	Ntl	2L:7711019-7714032	2.03	5.0000E-05	2.7364E-04
192	dpr16	dpr16	3R:976629-995849	2.03	5.0000E-05	2.7364E-04
193	fd96Cb	fd96Cb	3R:20920830-20921655	2.03	5.0000E-05	2.7364E-04
194	rgn	rgn	3L:21375623-21426267	2.02	5.0000E-05	2.7364E-04
195	CG32557	CG32557	X:17746990-17750156	2.01	1.7000E-03	6.1650E-03
196	SP1173	SP1173	3L:6682424-6696619	2.00	5.0000E-05	2.7364E-04
197	CG5953	CG5953	2L:16509119-16532877	1.98	5.0000E-05	2.7364E-04
198	CG13177	CG13177	2R:8051179-8051898	1.98	7.1000E-03	2.0705E-02
199	dp	dp	2L:4479470-4591963	1.97	5.0000E-05	2.7364E-04
200	CG7800	CG7800	3R:4043699-4045456	1.97	5.0000E-05	2.7364E-04
201	CG1718	CG1718	X:21163740-21171389	1.97	5.0000E-05	2.7364E-04
202	CG31028	CG31028	3R:25992352-25996925	1.96	5.0000E-05	2.7364E-04
203	CG2269	CG2269	2R:5975075-5983921	1.96	5.0000E-05	2.7364E-04
204	CG14606	CG14606	3R:3040060-3042805	1.96	7.0000E-04	2.8502E-03
205	CG1623	CG1623	2R:5715605-5724147	1.96	1.5000E-04	7.3812E-04
206	CG8564	CG8564	3L:7378747-7380499	1.96	1.1500E-03	4.4143E-03
207	B-H2	B-H2	X:17208613-17218195	1.96	5.0000E-05	2.7364E-04
208	CG42730	CG42730	2L:6127871-6130659	1.96	5.0000E-04	2.1172E-03
209	CG4678	CG4678	X:16590164-16605178	1.96	5.0000E-05	2.7364E-04

210	upd3	upd3	X:18171265-18178632	1.96	5.0000E-05	2.7364E-04
211	bru-2	bru-2	2L:12344380-12397534	1.95	5.0000E-05	2.7364E-04
212	CG30377	CG30377	2R:3788457-3802515	1.95	5.0000E-05	2.7364E-04
213	CG10550	CG10550	3R:21132128-21134109	1.95	5.0000E-05	2.7364E-04
214	CG6643	CG6643	3R:20370440-20382271	1.94	5.0000E-05	2.7364E-04
215	CG32815	CG32815	X:824963-828792	1.92	3.0000E-04	1.3573E-03
216	Tsp42En	Tsp42En	2R:2940278-2941548	1.92	3.4500E-03	1.1176E-02
217	Syn2	Syn2	2R:12439764-12445988	1.91	5.0000E-05	2.7364E-04
218	Prestin	Prestin	3L:17892368-17894853	1.91	5.0000E-05	2.7364E-04
219	CG7349	CG7349	X:18788539-18790616	1.91	5.0000E-05	2.7364E-04
220	CG7742	CG7742	2L:5210460-5214730	1.91	3.3500E-03	1.0890E-02
221	CG8925	CG8925	3R:11997730-12006200	1.91	5.0000E-05	2.7364E-04
222	nvy	nvy	2R:20163259-20178611	1.91	5.0000E-05	2.7364E-04
223	CG5928	CG5928	X:5961992-5968890	1.91	1.1700E-02	3.1534E-02
224	CG33225	CG33225	2R:17477890-17479089	1.90	5.0000E-05	2.7364E-04
225	CG42808	CG42808	2R:9461122-9461747	1.89	8.0000E-04	3.2221E-03
226	Act57B	Act57B	2R:16831532-16833945	1.89	1.7000E-03	6.1650E-03
227	jdp	jdp	3R:26342773-26352761	1.89	5.0000E-05	2.7364E-04
228	LvpD	LvpD	2R:4341136-4343291	1.89	5.0000E-05	2.7364E-04
229	CG8547	CG8547	2R:10148710-10155419	1.89	5.0000E-05	2.7364E-04
230	Sox15	Sox15	2R:10088887-10099914	1.89	5.0000E-05	2.7364E-04
231	CG5565	CG5565	2L:1352969-1355924	1.88	3.5000E-03	1.1303E-02
232	CG14709	CG14709	3R:7394971-7404467	1.87	5.0000E-05	2.7364E-04
233	CG1143	CG1143	3L:2645375-2646605	1.87	5.0000E-05	2.7364E-04
234	CG31755	CG31755	2L:9995560-10002446	1.87	5.0000E-05	2.7364E-04
235	CG1572	CG1572	X:11449317-11452687	1.87	5.0000E-05	2.7364E-04
236	CG13936	CG13936	3L:1850914-1855591	1.87	2.7500E-03	9.2394E-03
237	CG13624	CG13624	3R:20385001-20408577	1.86	5.0000E-05	2.7364E-04
238	Task7	Task7	3R:5236050-5238015	1.86	5.0000E-05	2.7364E-04
239	CG31233	CG31233	3R:17575574-17578959	1.86	5.0000E-05	2.7364E-04
240	CG6231	CG6231	3R:15441745-15458440	1.86	5.0000E-05	2.7364E-04
241	Cht4	Cht4	2R:16952884-16954592	1.85	9.5000E-04	3.7311E-03
242	CG15701	CG15701	2R:12110718-12121591	1.85	2.0000E-04	9.4764E-04
243	CG15459	CG15459	X:20283367-20284635	1.85	6.9500E-03	2.0318E-02
244	CG32262	CG32262	3L:3809145-3810181	1.85	1.5600E-02	3.9888E-02
245	PebIII	PebIII	2R:19914055-19915040	1.84	5.0000E-05	2.7364E-04
246	CG14275	CG14275	2L:8327457-8332911	1.84	5.0000E-05	2.7364E-04
247	CG9119	CG9119	3L:1203315-1204792	1.84	8.1500E-03	2.3208E-02
248	CG13856	CG13856	3R:18228376-18230783	1.84	7.8000E-03	2.2396E-02
249	Rcd2	Rcd2	3L:20521067-20533514	1.83	5.0000E-05	2.7364E-04
250	CG9313	CG9313	2R:16866848-16874000	1.83	5.0000E-05	2.7364E-04
251	CG9455	CG9455	2R:2769169-2772378	1.82	5.0000E-05	2.7364E-04
252	CG11835	CG11835	2L:560793-563303	1.82	5.0000E-05	2.7364E-04

253	chp	chp	3R:27029903-27036452	1.82	5.0000E-05	2.7364E-04
254	CG15414	CG15414	2L:3480754-3491048	1.81	5.0000E-05	2.7364E-04
255	CG4562	CG4562	3R:15692861-15700941	1.81	5.0000E-05	2.7364E-04
256	Tob	Tob	X:15973046-15985369	1.80	5.0000E-05	2.7364E-04
257	CG30460	CG30460	2R:12950281-12968762	1.80	5.0000E-05	2.7364E-04
258	CG34002	CG34002	3L:17851573-17853582	1.79	1.2000E-03	4.5893E-03
259	Tsp42El	Tsp42El	2R:2933277-2936050	1.79	5.0000E-05	2.7364E-04
260	GstD8	GstD8	3R:8205744-8206537	1.79	3.6000E-03	1.1558E-02
261	CG11841	CG11841	3R:24876777-24877989	1.78	3.7000E-03	1.1838E-02
262	Btd	Btd	X:6111512-6113327	1.78	6.5000E-04	2.6711E-03
263	CG11395	CG11395	2R:13024745-13026337	1.78	1.5000E-04	7.3812E-04
264	CG6372	CG6372	3L:8610334-8612574	1.77	5.0000E-05	2.7364E-04
265	per	per	X:2579612-2587919	1.77	5.0000E-05	2.7364E-04
266	CG31871	CG31871	2L:10662086-10671489	1.76	1.5000E-04	7.3812E-04
267	pdm3	pdm3	2R:4215004-4283785	1.75	5.0000E-05	2.7364E-04
268	Nox	Nox	2R:12197071-12203725	1.75	5.0000E-05	2.7364E-04
269	Socs36E	Socs36E	2L:18138667-18152410	1.75	5.0000E-05	2.7364E-04
270	NLaz	NLaz	2L:1359982-1361732	1.75	3.0500E-03	1.0095E-02
271	CG10483	CG10483	3L:5911004-5914398	1.75	2.0000E-04	9.4764E-04
272	CG10249	CG10249	2R:10788974-10815585	1.75	5.0000E-05	2.7364E-04
273	CG14566	CG14566	3L:21732578-21733409	1.74	1.0000E-04	5.1606E-04
274	Cht12	Cht12	2R:16949150-16952491	1.74	1.4850E-02	3.8317E-02
275	GstD6	GstD6	3R:8202893-8203629	1.74	5.0000E-05	2.7364E-04
276	CG7991	CG7991	3L:1674708-1715541	1.74	5.0000E-05	2.7364E-04
277	GstD4	GstD4	3R:8199823-8200546	1.73	5.0000E-05	2.7364E-04
278	CG5397	CG5397	2L:1240037-1242205	1.72	1.0000E-04	5.1606E-04
279	CG14225	CG14225	X:19566668-19569992	1.72	5.0000E-05	2.7364E-04
280	ry	ry	3R:8858258-8863748	1.71	5.0000E-05	2.7364E-04
281	Drep-3	Drep-3	2R:7940448-7950374	1.71	5.0000E-05	2.7364E-04
282	CG17930	CG17930	3R:12153571-12155345	1.71	3.4000E-03	1.1033E-02
283	CG9899	CG9899	2R:19046428-19049553	1.71	5.0000E-05	2.7364E-04
284	CG13893	CG13893	3L:593245-604001	1.71	5.0000E-05	2.7364E-04
285	CG2650	CG2650	X:2579612-2587919	1.70	1.3250E-02	3.4914E-02
286	CG5561	CG5561	2L:1352969-1355924	1.70	1.0350E-02	2.8438E-02
287	CG30456	CG30456	2R:12990371-12995047	1.70	3.4000E-03	1.1033E-02
288	Peritrophin-A	Peritrophin-A	X:20115726-20119273	1.70	5.0000E-05	2.7364E-04
289	yellow-e3	yellow-e3	3R:9229144-9230590	1.70	5.0000E-05	2.7364E-04
290	CG14955	CG14955	3L:3110127-3114248	1.69	5.0000E-05	2.7364E-04
291	os	os	X:18199376-18203021	1.69	5.0000E-05	2.7364E-04
292	Or7a	Or7a	X:8058298-8059741	1.69	3.9000E-03	1.2401E-02
293	CG5455	CG5455	3R:22287250-22295732	1.69	5.0000E-05	2.7364E-04
294	CG8066	CG8066	3R:10393331-10394532	1.68	1.5000E-04	7.3812E-04

295	CG9454	CG9454	2R:2766822-2768220	1.68	2.1500E-03	7.5077E-03
296	CG31002	CG31002	3R:27219303-27221020	1.68	1.4000E-03	5.2360E-03
297	nemy	nemy	2R:8557987-8567094	1.67	5.0000E-05	2.7364E-04
298	Act87E	Act87E	3R:9251706-9253811	1.67	5.0000E-05	2.7364E-04
299	CG31036	CG31036	3R:25733830-25738614	1.67	5.0000E-05	2.7364E-04
300	Ahcy89E	Ahcy89E	3R:12804160-12807905	1.67	3.5000E-04	1.5496E-03
301	Ilp6	Ilp6	X:2225525-2227719	1.67	1.2500E-03	4.7454E-03
302	CG13032	CG13032	3L:16667063-16669364	1.66	1.0000E-04	5.1606E-04
303	CG13183	CG13183	2R:7791827-7800280	1.66	5.0000E-05	2.7364E-04
304	CG31324	CG31324	3R:21932591-21955912	1.66	2.3000E-03	7.9393E-03
305	CG32392	CG32392	3L:6749722-6756346	1.65	5.0000E-05	2.7364E-04
306	CG12934	CG12934	2R:6536387-6537250	1.65	3.0000E-04	1.3573E-03
307	Drip	Drip	2R:7299446-7317146	1.65	5.0000E-05	2.7364E-04
308	CG8630	CG8630	3R:9105444-9110316	1.65	2.0000E-04	9.4764E-04
309	CG32703	CG32703	X:9155408-9161731	1.64	5.0000E-05	2.7364E-04
310	CG3078	CG3078	X:2148431-2153896	1.64	5.0000E-05	2.7364E-04
311	btl	btl	3L:14064991-14075985	1.64	5.0000E-05	2.7364E-04
312	CG17667	CG17667	3L:12777528-12798214	1.64	3.5500E-03	1.1437E-02
313	m6	m6	3R:21858632-21859769	1.64	5.0000E-05	2.7364E-04
314	Gr59e	Gr59e	2R:19428576-19429902	1.63	1.5000E-03	5.5542E-03
315	CG9447	CG9447	2R:2760836-2763118	1.63	5.0000E-04	2.1172E-03
316	fend	fend	X:9019164-9029051	1.63	5.0000E-05	2.7364E-04
317	CG42235	CG42235	3R:21732751-21748219	1.62	4.5000E-04	1.9354E-03
318	CG42755	CG42755	3L:11376483-11377323	1.62	2.0050E-02	4.9344E-02
319	CG15005	CG15005	3L:4193992-4204338	1.62	1.3650E-02	3.5796E-02
320	CG7886	CG7886	3R:10452394-10468285	1.62	5.0000E-05	2.7364E-04
321	Cpr62Bc	Cpr62Bc	3L:1840277-1842080	1.62	1.6300E-02	4.1451E-02
322	CG7884	CG7884	X:19114656-19129946	1.61	5.0000E-05	2.7364E-04
323	ppk12	ppk12	2R:18314983-18317089	1.61	2.9000E-03	9.6632E-03
324	Oatp33Ea	Oatp33Ea	2L:12441399-12445911	1.61	6.5000E-04	2.6711E-03
325	kay	kay	3R:25592496-25619838	1.61	5.0000E-05	2.7364E-04
326	Tsp42Ed	Tsp42Ed	2R:2897364-2899359	1.61	2.8000E-03	9.3904E-03
327	burs	burs	3R:17595272-17595916	1.60	1.6750E-02	4.2364E-02
328	yellow-b	yellow-b	2L:16754335-16758283	1.60	5.0000E-05	2.7364E-04
329	CG11872	CG11872	3R:6128611-6133442	1.60	5.0000E-05	2.7364E-04
330	Hsc70-1	Hsc70-1	3L:13967276-13970879	1.60	5.0000E-05	2.7364E-04
331	Edg91	Edg91	3R:13436577-13437233	1.59	5.0000E-05	2.7364E-04
332	CG15611	CG15611	2R:12996351-13001866	1.59	5.0000E-05	2.7364E-04
333	CG6967	CG6967	2R:12950281-12968762	1.59	5.1500E-03	1.5742E-02
334	Tsp33B	Tsp33B	2L:11791024-11792365	1.58	3.5000E-04	1.5496E-03
335	CG8028	CG8028	X:19171288-19174381	1.58	1.5000E-03	5.5542E-03
336	CG6999	CG6999	X:8982399-8983347	1.57	3.5000E-04	1.5496E-03
337	CG10126	CG10126	3R:8681259-8684939	1.56	5.0000E-05	2.7364E-04

338	AdoR	AdoR	3R:25960996-25964247	1.56	1.1500E-03	4.4143E-03
339	LvpH	LvpH	2R:4334973-4339404	1.56	3.6000E-03	1.1558E-02
340	yellow-f2	yellow-f2	3R:8819114-8820869	1.56	5.0000E-05	2.7364E-04
341	ort	ort	3R:15485469-15489401	1.56	2.3000E-03	7.9393E-03
342	ppk23	ppk23	X:17460818-17463168	1.56	1.8000E-03	6.4647E-03
343	Cpr57A	Cpr57A	2R:16435871-16437597	1.56	5.0000E-05	2.7364E-04
344	Or9a	Or9a	X:10352218-10353709	1.56	2.0000E-04	9.4764E-04
345	su(r)	su(r)	X:9090838-9096770	1.55	9.5000E-04	3.7311E-03
346	mwh	mwh	3L:1208424-1232701	1.55	5.0000E-05	2.7364E-04
347	ken	ken	2R:19757797-19764965	1.55	5.0000E-05	2.7364E-04
348	gd	gd	X:11879566-11881780	1.55	8.0000E-04	3.2221E-03
349	CG32301	CG32301	3L:2244529-2248483	1.55	5.0000E-05	2.7364E-04
350	CG9722	CG9722	3R:9978606-9979551	1.55	1.7350E-02	4.3727E-02
351	tup	tup	2L:18859499-18881256	1.55	5.0000E-05	2.7364E-04
352	CG1441	CG1441	2R:5786522-5790788	1.55	5.0000E-05	2.7364E-04
353	CG42575	CG42575	3L:11116460-11127763	1.55	5.0000E-05	2.7364E-04
354	CG10560	CG10560	3R:21144271-21145801	1.54	5.0000E-05	2.7364E-04
355	CG2196	CG2196	3R:27577546-27580413	1.54	5.0000E-05	2.7364E-04
356	hdc	hdc	3R:26103655-26187891	1.54	5.0000E-05	2.7364E-04
357	CG31897	CG31897	2L:8442446-8445660	1.54	5.0000E-05	2.7364E-04
358	CG17639	CG17639	3R:8209082-8211348	1.54	9.0500E-03	2.5299E-02
359	CG8394	CG8394	2R:10086504-10088226	1.53	5.0000E-05	2.7364E-04
360	SNF4Agamma	SNF4Agamma	3R:16966462-17038409	1.53	5.0000E-05	2.7364E-04
361	dyl	dyl	3L:4293994-4307711	1.53	5.0000E-05	2.7364E-04
362	zfh2	zfh2	4:524476-560418	1.53	5.0000E-05	2.7364E-04
363	ab	ab	2L:11210680-11269179	1.53	5.0000E-05	2.7364E-04
364	Mef2	Mef2	2R:5801000-5846313	1.53	5.0000E-05	2.7364E-04
365	CG8709	CG8709	2R:4024823-4044128	1.53	5.0000E-05	2.7364E-04
366	lectin-28C	lectin-28C	2L:7857089-7857931	1.53	7.3000E-03	2.1215E-02
367	CG3323	CG3323	X:5190113-5201450	1.52	5.2000E-03	1.5864E-02
368	Or65a	Or65a	3L:6313710-6315198	1.52	1.5500E-03	5.6919E-03
369	CG3706	CG3706	X:824963-828792	1.52	3.2000E-03	1.0487E-02
370	Cha	Cha	3R:14531173-14558068	1.52	5.0000E-05	2.7364E-04
371	CG3841	CG3841	2L:9589475-9591783	1.52	6.2000E-03	1.8461E-02
372	CG30345	CG30345	2R:5036331-5038809	1.51	3.0000E-04	1.3573E-03
373	CG31547	CG31547	3R:1315226-1328424	1.51	5.0000E-05	2.7364E-04
374	CG42249	CG42249	X:10990305-11002192	1.51	4.8500E-03	1.5012E-02
375	CG7059	CG7059	3R:18215501-18219826	1.51	2.0000E-04	9.4764E-04
376	CG31875	CG31875	2L:9995560-10002446	1.51	2.0500E-03	7.2098E-03
377	form3	form3	3L:7087917-7118623	1.51	5.0000E-05	2.7364E-04
378	CG17669	CG17669	2R:14340241-14341967	1.51	5.0000E-05	2.7364E-04
379	CG7227	CG7227	2L:7994836-7998144	1.51	1.9500E-03	6.9182E-03

380	Cyp12a5	Cyp12a5	3R:14958173-14960429	1.50	5.0000E-05	2.7364E-04
381	CG1674	CG1674	4:251355-266529	1.50	5.0000E-05	2.7364E-04
382	CG6043	CG6043	2L:13261009-13286409	1.50	5.0000E-05	2.7364E-04
383	Ance-2	Ance-2	2L:13909639-13911640	1.50	5.0000E-05	2.7364E-04
384	kirre	kirre	X:2846239-3026836	1.49	6.5000E-04	2.6711E-03
385	CG13995	CG13995	2L:6054306-6060169	1.49	5.0000E-04	2.1172E-03
386	CG31676	CG31676	2L:20840799-20849182	1.49	3.5000E-04	1.5496E-03
387	CG2254	CG2254	X:7972381-7975635	1.49	1.3650E-02	3.5796E-02
388	CG42636	CG42636	3L:19712626-19786663	1.48	1.0000E-04	5.1606E-04
389	CG10253	CG10253	2R:10817587-10822366	1.48	4.0000E-04	1.7372E-03
390	CG10226	CG10226	3L:6227416-6232689	1.48	5.0000E-05	2.7364E-04
391	CAH2	CAH2	3L:12172978-12176455	1.48	5.0000E-05	2.7364E-04
392	CG4267	CG4267	2L:2242378-2244415	1.48	5.0000E-05	2.7364E-04
393	CG13300	CG13300	3L:6324129-6325996	1.48	5.0000E-05	2.7364E-04
394	pwn	pwn	2R:3182812-3187907	1.48	5.0000E-05	2.7364E-04
395	peb	peb	X:4511601-4520256	1.48	5.0000E-05	2.7364E-04
396	CG12643	CG12643	X:10158256-10159290	1.48	5.0000E-05	2.7364E-04
397	CG14441	CG14441	X:6593878-6611012	1.47	5.0000E-05	2.7364E-04
398	Cyp28c1	Cyp28c1	X:11733640-11735414	1.47	1.3450E-02	3.5351E-02
399	CG14329	CG14329	3R:13435083-13436255	1.47	2.2000E-03	7.6508E-03
400	trpml	trpml	3L:19706959-19711450	1.47	1.5000E-04	7.3812E-04
401	CG4907	CG4907	3R:18533507-18536485	1.47	1.7500E-03	6.3168E-03
402	CG3884	CG3884	2R:8835852-8839791	1.47	5.5000E-04	2.3101E-03
403	CG4096	CG4096	X:5593219-5611811	1.47	5.0000E-05	2.7364E-04
404	CG5070	CG5070	X:17097146-17097958	1.46	4.7500E-03	1.4727E-02
405	CG14984	CG14984	3L:3951189-3952420	1.46	5.0000E-05	2.7364E-04
406	CG1124	CG1124	3R:791168-794226	1.45	9.9500E-03	2.7501E-02
407	Lsp1beta	Lsp1beta	2L:898646-901320	1.45	5.0000E-05	2.7364E-04
408	CG7402	CG7402	3L:17880804-17884504	1.45	1.8500E-03	6.6214E-03
409	CG8177	CG8177	3L:9756651-9779365	1.45	5.0000E-05	2.7364E-04
410	fz3	fz3	X:664060-677344	1.44	5.0000E-05	2.7364E-04
411	CG30181	CG30181	2R:19426153-19428296	1.44	6.4500E-03	1.9102E-02
412	sls	sls	3L:2040178-2115617	1.44	5.0000E-05	2.7364E-04
413	cv-c	cv-c	3R:10217401-10307410	1.43	5.0000E-05	2.7364E-04
414	CG13928	CG13928	3L:1618391-1629298	1.43	4.0000E-04	1.7372E-03
415	dpp	dpp	2L:2428453-2459609	1.43	5.0000E-05	2.7364E-04
416	CG32364	CG32364	3L:8202676-8203771	1.43	5.0000E-05	2.7364E-04
417	disco-r	disco-r	X:16012820-16042982	1.43	5.0000E-05	2.7364E-04
418	CG34265	CG34265	3L:3268494-3268994	1.42	4.0000E-04	1.7372E-03
419	dpr17	dpr17	3R:7920599-7937144	1.42	5.0000E-05	2.7364E-04
420	CG11155	CG11155	4:1147232-1156547	1.42	5.0000E-05	2.7364E-04
421	CG2736	CG2736	2R:20860943-20862894	1.42	5.0000E-05	2.7364E-04
422	Arc1	Arc1	2R:10245427-10247799	1.42	5.0000E-05	2.7364E-04

423	gsb	gsb	2R:20949501-20952166	1.42	2.2000E-03	7.6508E-03
424	CG12268	CG12268	3R:19769349-19774141	1.42	5.0000E-05	2.7364E-04
425	CG31087	CG31087	3R:21136748-21139555	1.41	1.2200E-02	3.2571E-02
426	ckd	ckd	3L:3258154-3267845	1.41	5.0000E-05	2.7364E-04
427	CG30497	CG30497	2R:3625272-3670119	1.41	5.0000E-05	2.7364E-04
428	Cyp6g2	Cyp6g2	2R:8073167-8077882	1.41	2.7000E-03	9.0910E-03
429	CG31776	CG31776	2L:3470181-3472368	1.41	5.0000E-04	2.1172E-03
430	Ptr	Ptr	2R:2069479-2083512	1.40	5.0000E-05	2.7364E-04
431	CG6428	CG6428	X:3859458-3862597	1.40	1.5000E-04	7.3812E-04
432	Rph	Rph	X:10643109-10651699	1.40	5.0000E-05	2.7364E-04
433	CG8083	CG8083	2R:4966231-4969879	1.40	4.0000E-04	1.7372E-03
434	Gr93d	Gr93d	3R:17671175-17672381	1.40	1.5000E-04	7.3812E-04
435	Ccap	Ccap	3R:18526303-18530061	1.39	5.2000E-03	1.5864E-02
436	Dhc62B	Dhc62B	3L:1756158-1771650	1.39	1.1850E-02	3.1855E-02
437	Cyp4e1	Cyp4e1	2R:4334973-4339404	1.39	5.0000E-05	2.7364E-04
438	CG6490	CG6490	3R:22296824-22360939	1.38	5.0000E-05	2.7364E-04
439	CG13003	CG13003	X:16738839-16747269	1.38	5.0000E-05	2.7364E-04
440	Strn-Mlck	Strn-Mlck	2R:11830385-11873458	1.38	5.0000E-05	2.7364E-04
441	tio	tio	2L:22019315-22024313	1.38	5.0000E-05	2.7364E-04
442	CG2187	CG2187	3R:27581077-27584359	1.38	3.5000E-04	1.5496E-03
443	CG13299	CG13299	3L:6224904-6225461	1.37	9.0500E-03	2.5299E-02
444	CG11321	CG11321	2L:6760526-6770949	1.37	3.0000E-04	1.3573E-03
445	C901	C901	X:10858239-10861165	1.37	1.2000E-03	4.5893E-03
446	Fmo-1	Fmo-1	2R:19850497-19852175	1.37	8.5500E-03	2.4103E-02
447	itp	itp	2R:20419122-20446273	1.37	5.0000E-05	2.7364E-04
448	Esp	Esp	3R:20667764-20676134	1.37	3.9500E-03	1.2526E-02
449	GstD5	GstD5	3R:8201485-8202136	1.37	3.5000E-04	1.5496E-03
450	Ance-5	Ance-5	2R:20776150-20778545	1.37	5.0000E-05	2.7364E-04
451	Cht6	Cht6	X:9924144-9938633	1.37	5.0000E-05	2.7364E-04
452	CG8398	CG8398	3L:6602424-6611280	1.37	2.5000E-04	1.1552E-03
453	CG13036	CG13036	3L:16384221-16384781	1.36	4.2500E-03	1.3364E-02
454	RhoGAP100F	RhoGAP100F	3R:27638796-27670966	1.36	5.0000E-05	2.7364E-04
455	osp	osp	2L:14599775-14689325	1.36	5.0000E-05	2.7364E-04
456	ss	ss	3R:12200147-12229405	1.36	5.0000E-05	2.7364E-04
457	Dr	Dr	3R:25382108-25391007	1.36	5.0000E-05	2.7364E-04
458	DAT	DAT	2R:12446061-12452763	1.36	5.0000E-05	2.7364E-04
459	Ddr	Ddr	2L:6253122-6322251	1.36	5.0000E-05	2.7364E-04
460	CG42335	CG42335	3R:17584495-17595023	1.36	1.0000E-04	5.1606E-04
461	lmd	lmd	3R:18846943-18852435	1.36	5.0000E-05	2.7364E-04
462	cry	cry	3R:15037914-15041166	1.35	9.5000E-04	3.7311E-03
463	puc	puc	3R:3931056-3948023	1.35	5.0000E-05	2.7364E-04
464	disco	disco	X:16104630-16110767	1.35	5.0000E-05	2.7364E-04

465	Gr59f	Gr59f	2R:19430032-19431408	1.35	2.5500E-03	8.6706E-03
466	CG14949	CG14949	3L:2642183-2643424	1.35	1.0000E-04	5.1606E-04
467	CG15117	CG15117	2R:15009734-15016746	1.35	3.7000E-03	1.1838E-02
468	Cpr64Ad	Cpr64Ad	3L:4215386-4216414	1.35	2.7000E-03	9.0910E-03
469	CG17991	CG17991	3R:23066988-23068052	1.35	1.4500E-03	5.3927E-03
470	CG34043	CG34043	2L:10278950-10280846	1.34	7.8000E-03	2.2396E-02
471	CG11293	CG11293	2R:19573201-19575503	1.34	1.0250E-02	2.8222E-02
472	CG42637	CG42637	3L:19712626-19786663	1.34	2.5000E-04	1.1552E-03
473	stg	stg	3R:25077539-25081502	1.34	5.0000E-05	2.7364E-04
474	CG8100	CG8100	3L:14076727-14078834	1.34	5.0000E-05	2.7364E-04
475	Phlpp	Phlpp	2L:19059646-19063049	1.34	5.0000E-05	2.7364E-04
476	CG15196	CG15196	X:11303979-11305700	1.34	5.0000E-05	2.7364E-04
477	neur	neur	3R:4846067-4868072	1.34	5.0000E-05	2.7364E-04
478	CG8908	CG8908	2R:16152436-16158290	1.34	5.0000E-05	2.7364E-04
479	dac	dac	2L:16466509-16485984	1.34	5.0000E-05	2.7364E-04
480	ths	ths	2R:7677873-7699520	1.34	5.0000E-05	2.7364E-04
481	ttm2	ttm2	3L:275163-276661	1.33	1.2350E-02	3.2905E-02
482	tld	tld	3R:20575455-20579234	1.33	5.0000E-05	2.7364E-04
483	Faa	Faa	3L:4072031-4073949	1.33	2.0000E-04	9.4764E-04
484	CG11381	CG11381	X:1097811-1099468	1.33	7.5000E-03	2.1681E-02
485	CG15093	CG15093	2R:14663173-14664481	1.33	5.0000E-05	2.7364E-04
486	CG14567	CG14567	3L:21730704-21731530	1.33	5.0000E-05	2.7364E-04
487	Orct	Orct	3R:20101981-20104515	1.33	5.0000E-05	2.7364E-04
488	CG13771	CG13771	2L:6662059-6663614	1.33	5.5000E-04	2.3101E-03
489	CG13891	CG13891	3L:433145-433906	1.32	1.9900E-02	4.9040E-02
490	CG3759	CG3759	2L:9416592-9426342	1.32	2.3000E-03	7.9393E-03
491	CG10936	CG10936	2R:13492455-13526639	1.32	5.0000E-05	2.7364E-04
492	CG12065	CG12065	X:8468336-8482561	1.32	5.0000E-05	2.7364E-04
493	CG31792	CG31792	2L:18997994-19002483	1.31	5.0000E-05	2.7364E-04
494	CG12964	CG12964	2R:11373456-11384712	1.31	5.0000E-05	2.7364E-04
495	pncr003:2L	pncr003:2L	2L:16838592-16841887	1.31	2.3000E-03	7.9393E-03
496	CG8620	CG8620	3L:7151198-7152010	1.31	1.1500E-03	4.4143E-03
497	CG32521	CG32521	X:21076336-21152198	1.31	5.0000E-05	2.7364E-04
498	pxb	pxb	3R:11491907-11513658	1.31	5.0000E-05	2.7364E-04
499	CG13692	CG13692	2L:421799-422435	1.31	2.9000E-03	9.6632E-03
500	GstD7	GstD7	3R:8204260-8204977	1.31	2.5500E-03	8.6706E-03
501	pros	pros	3R:7197708-7219326	1.31	5.0000E-05	2.7364E-04
502	PHDP	PHDP	2R:19770707-19771835	1.31	3.6000E-03	1.1558E-02
503	CG1698	CG1698	2R:5653866-5694370	1.30	5.0000E-05	2.7364E-04
504	CG7016	CG7016	3R:20688653-20690502	1.30	5.0000E-04	2.1172E-03
