# Supplementary material for: The Drosophila Duox maturation factor is a key component of a positive feedback loop that sustains regeneration signaling
Source: PLoS Genet. 2017 Jul 28;13(7):e1006937. doi: 10.1371/journal.pgen.1006937 (PMC5550008; doi:10.1371/journal.pgen.1006937)
Supplement: S2 Table — (PDF) [file pgen.1006937.s010.pdf]

**Table S2: List of Downregulated genes (log2 fold change ≤ -1.3, p<0.05)**

| # | gene_id | gene | locus | log2(fold _change) | p_value | q_value |
|---|---|---|---|---|---|---|
| 1 | Osi1 | Osi1 | 3R:1999366-2000555 | -inf | 5.0000E-05 | 2.7364E-04 |
| 2 | Obp46a | Obp46a | 2R:6198676-6199528 | -6.14 | 2.6500E-03 | 8.9486E-03 |
| 3 | Optix | Optix | 2R:3919137-3930041 | -5.96 | 5.0000E-05 | 2.7364E-04 |
| 4 | Hr46 | Hr46 | 2R:6092958-6124853 | -5.52 | 5.0000E-05 | 2.7364E-04 |
| 5 | CG32372 | CG32372 | 3L:7512587-7521116 | -5.40 | 5.0000E-05 | 2.7364E-04 |
| 6 | Tsp42Ei | Tsp42Ei | 2R:2922631-2925174 | -4.92 | 5.0000E-05 | 2.7364E-04 |
| 7 | CG1139 | CG1139 | 3L:1971661-1974285 | -4.73 | 5.0000E-05 | 2.7364E-04 |
| 8 | CG16884 | CG16884 | 2L:13914724-13955757 | -4.64 | 2.0000E-04 | 9.4764E-04 |
| 9 | NetA | NetA | X:14498044-14547864 | -4.61 | 5.0000E-05 | 2.7364E-04 |
| 10 | CG42527 | CG42527 | X:1891619-1898128 | -4.58 | 5.0000E-05 | 2.7364E-04 |
| 11 | Poxn | Poxn | 2R:11714197-11722280 | -4.53 | 5.0000E-05 | 2.7364E-04 |
| 12 | CG14915 | CG14915 | 2L:11071026-11071532 | -4.44 | 5.0000E-05 | 2.7364E-04 |
| 13 | CG1342 | CG1342 | 3R:26516365-26518490 | -4.36 | 5.0000E-05 | 2.7364E-04 |
| 14 | sm | sm | 2R:15417413-15519007 | -4.34 | 5.0000E-05 | 2.7364E-04 |
| 15 | Cpr78E | Cpr78E | 3L:21659553-21660080 | -4.19 | 5.0000E-05 | 2.7364E-04 |
| 16 | Lkr | Lkr | 3L:5505516-5522441 | -4.14 | 9.0000E-04 | 3.5694E-03 |
| 17 | ImpE2 | ImpE2 | 3L:3510973-3512677 | -3.97 | 5.0000E-05 | 2.7364E-04 |
| 18 | ana | ana | 2R:4956605-4965059 | -3.65 | 5.0000E-05 | 2.7364E-04 |
| 19 | Blimp-1 | Blimp-1 | 3L:5624082-5642049 | -3.57 | 5.0000E-05 | 2.7364E-04 |
| 20 | CG8483 | CG8483 | 3R:9034469-9040187 | -3.50 | 5.0000E-05 | 2.7364E-04 |
| 21 | CG10205 | CG10205 | 2R:10720337-10721460 | -3.47 | 2.0000E-04 | 9.4764E-04 |
| 22 | hoe1 | hoe1 | 2L:4908744-4932296 | -3.43 | 5.0000E-05 | 2.7364E-04 |
| 23 | CG16733 | CG16733 | 3R:5077574-5078687 | -3.37 | 5.0000E-05 | 2.7364E-04 |
| 24 | CG16885 | CG16885 | 2L:13914724-13955757 | -3.36 | 5.0000E-05 | 2.7364E-04 |
| 25 | CG13082 | CG13082 | 2L:19538428-19539910 | -3.35 | 5.0000E-05 | 2.7364E-04 |
| 26 | Doc2 | Doc2 | 3L:9005785-9012340 | -3.31 | 5.0000E-05 | 2.7364E-04 |
| 27 | CG8420 | CG8420 | 3R:5073172-5077160 | -3.30 | 5.0000E-05 | 2.7364E-04 |
| 28 | dve | dve | 2R:18131467-18173982 | -3.21 | 5.0000E-05 | 2.7364E-04 |
| 29 | CG31975 | CG31975 | 2L:138383-140992 | -3.15 | 5.0000E-05 | 2.7364E-04 |
| 30 | CG4586 | CG4586 | X:6863973-6866302 | -3.11 | 5.0000E-05 | 2.7364E-04 |
| 31 | Doc1 | Doc1 | 3L:9034476-9038144 | -3.00 | 5.0000E-05 | 2.7364E-04 |
| 32 | Wnt2 | Wnt2 | 2R:5381673-5390695 | -2.99 | 5.0000E-05 | 2.7364E-04 |
| 33 | CG7888 | CG7888 | 3L:11047136-11054319 | -2.95 | 3.0000E-04 | 1.3573E-03 |
| 34 | CG3823 | CG3823 | X:6198022-6200778 | -2.90 | 5.0000E-05 | 2.7364E-04 |
| 35 | CG7201 | CG7201 | 3L:8283385-8288422 | -2.88 | 5.0000E-05 | 2.7364E-04 |
| 36 | CG7367 | CG7367 | 2L:8026913-8039788 | -2.85 | 2.0100E-02 | 4.9415E-02 |
| 37 | CG31728 | CG31728 | 2L:13255407-13257426 | -2.82 | 5.0000E-05 | 2.7364E-04 |
| 38 | CG10029 | CG10029 | 3R:3141445-3142929 | -2.75 | 1.5000E-04 | 7.3812E-04 |
| 39 | ImpE3 | ImpE3 | 3R:3839545-3856505 | -2.72 | 5.0000E-05 | 2.7364E-04 |
| 40 | CG30080 | CG30080 | 2R:11586230-11589199 | -2.71 | 5.0000E-05 | 2.7364E-04 |
| 41 | CG15765 | CG15765 | X:5712596-5733606 | -2.66 | 5.0000E-05 | 2.7364E-04 |
| 42 | trh | trh | 3L:366523-392633 | -2.58 | 5.0000E-05 | 2.7364E-04 |
| 43 | CR42861 | CR42861 | X:13638960-13639509 | -2.58 | 5.0000E-05 | 2.7364E-04 |
| 44 | Os-C | Os-C | 3R:3804680-3806044 | -2.56 | 5.0000E-05 | 2.7364E-04 |
| 45 | CG30380 | CG30380 | 2R:3826705-3827144 | -2.56 | 2.6000E-03 | 8.7988E-03 |
| 46 | spz | spz | 3R:22890791-22895790 | -2.51 | 5.0000E-05 | 2.7364E-04 |
| 47 | al | al | 2L:378115-387439 | -2.50 | 5.0000E-05 | 2.7364E-04 |
| 48 | CG2930 | CG2930 | X:3766453-3774329 | -2.50 | 5.0000E-05 | 2.7364E-04 |

| | | | | | | |
|---|---|---|---|---|---|---|
| 49 | spirit | spirit | X:8465131-8467614 | -2.46 | 5.0000E-05 | 2.7364E-04 |
| 50 | Dhc93AB | Dhc93AB | 3R:16850740-16868141 | -2.42 | 5.0000E-05 | 2.7364E-04 |
| 51 | ppk13 | ppk13 | 2L:21086899-21088730 | -2.38 | 5.0000E-05 | 2.7364E-04 |
| 52 | Nep1 | Nep1 | X:5985802-5998006 | -2.36 | 5.0000E-05 | 2.7364E-04 |
| 53 | CG15546 | CG15546 | 3R:26700086-26701908 | -2.33 | 5.0000E-05 | 2.7364E-04 |
| 54 | CG33509 | CG33509 | 2L:21084604-21086506 | -2.33 | 5.0000E-05 | 2.7364E-04 |
| 55 | RpS19b | RpS19b | 3R:19758450-19759252 | -2.32 | 5.0000E-05 | 2.7364E-04 |
| 56 | CG14257 | CG14257 | 3R:22901604-22908543 | -2.31 | 5.0000E-05 | 2.7364E-04 |
| 57 | CG10960 | CG10960 | 3L:12817981-12836205 | -2.29 | 5.0000E-05 | 2.7364E-04 |
| 58 | CG9416 | CG9416 | 2R:15251149-15260747 | -2.26 | 5.0000E-05 | 2.7364E-04 |
| 59 | CG3568 | CG3568 | X:4436662-4438530 | -2.25 | 5.0000E-05 | 2.7364E-04 |
| 60 | CG13081 | CG13081 | 2L:19540692-19541343 | -2.24 | 5.0000E-05 | 2.7364E-04 |
| 61 | CG10026 | CG10026 | 2L:19454255-19457726 | -2.19 | 5.0000E-05 | 2.7364E-04 |
| 62 | Cpr49Ac | Cpr49Ac | 2R:8274471-8277562 | -2.19 | 5.0000E-05 | 2.7364E-04 |
| 63 | CG15905 | CG15905 | 2R:15533987-15535150 | -2.18 | 5.0000E-05 | 2.7364E-04 |
| 64 | CG3355 | CG3355 | 2L:4651402-4652877 | -2.17 | 5.0000E-05 | 2.7364E-04 |
| 65 | wbl | wbl | 2R:15137265-15138419 | -2.17 | 5.0000E-05 | 2.7364E-04 |
| 66 | Inos | Inos | 2R:3342418-3346368 | -2.16 | 5.0000E-05 | 2.7364E-04 |
| 67 | Mur89F | Mur89F | 3R:12979241-12985670 | -2.15 | 5.0000E-05 | 2.7364E-04 |
| 68 | CG13258 | CG13258 | 2L:16292126-16292970 | -2.15 | 5.0000E-05 | 2.7364E-04 |
| 69 | CG17278 | CG17278 | 3R:16846755-16849885 | -2.12 | 5.0000E-05 | 2.7364E-04 |
| 70 | CG12551 | CG12551 | 2R:1808437-1808752 | -2.11 | 2.9500E-03 | 9.8088E-03 |
| 71 | Cpr97Ea | Cpr97Ea | 3R:22908853-22912341 | -2.03 | 6.5000E-04 | 2.6711E-03 |
| 72 | CG5873 | CG5873 | 3R:13251493-13272215 | -2.01 | 5.0000E-05 | 2.7364E-04 |
| 73 | snoRNA:Psi28S-3342 | snoRNA:Psi28S-3342 | X:5418334-5418498 | -2.00 | 2.0000E-04 | 9.4764E-04 |
| 74 | CG9427 | CG9427 | 3R:5450874-5452187 | -1.99 | 5.0000E-05 | 2.7364E-04 |
| 75 | kal-1 | kal-1 | 3R:19967116-19974645 | -1.96 | 5.0000E-05 | 2.7364E-04 |
| 76 | CG5697 | CG5697 | 3R:16841670-16842842 | -1.95 | 5.0000E-05 | 2.7364E-04 |
| 77 | Eip78C | Eip78C | 3L:21220018-21257721 | -1.94 | 5.0000E-05 | 2.7364E-04 |
| 78 | Cpr30F | Cpr30F | 2L:9932586-9933092 | -1.93 | 4.3000E-03 | 1.3516E-02 |
| 79 | CG3649 | CG3649 | 2R:18733523-18741220 | -1.93 | 5.0000E-05 | 2.7364E-04 |
| 80 | CG7720 | CG7720 | 3R:14584957-14620876 | -1.91 | 5.0000E-05 | 2.7364E-04 |
| 81 | Ugt86Di | Ugt86Di | 3R:6977628-6979571 | -1.90 | 5.0000E-05 | 2.7364E-04 |
| 82 | CG6055 | CG6055 | 2L:7466545-7472493 | -1.86 | 5.0000E-05 | 2.7364E-04 |
| 83 | CG32706 | CG32706 | X:8981161-8982109 | -1.85 | 5.0000E-05 | 2.7364E-04 |
| 84 | CG32581 | CG32581 | X:15690935-15693189 | -1.84 | 5.0000E-05 | 2.7364E-04 |
| 85 | CG14118 | CG14118 | 3L:12885757-12887858 | -1.84 | 5.0000E-05 | 2.7364E-04 |
| 86 | betaTub97EF | betaTub97EF | 3R:23792729-23814089 | -1.78 | 5.0000E-05 | 2.7364E-04 |
| 87 | Lsd-2 | Lsd-2 | X:14965488-14970236 | -1.78 | 5.0000E-05 | 2.7364E-04 |
| 88 | Rpt6R | Rpt6R | 3R:26407919-26409419 | -1.77 | 5.0000E-05 | 2.7364E-04 |
| 89 | t | t | X:9111687-9117290 | -1.77 | 5.0000E-05 | 2.7364E-04 |
| 90 | CG5639 | CG5639 | 3R:23408526-23419260 | -1.76 | 5.0000E-05 | 2.7364E-04 |
| 91 | Fkbp13 | Fkbp13 | 2R:17374669-17385431 | -1.76 | 5.0000E-05 | 2.7364E-04 |
| 92 | AnnX | AnnX | X:20064247-20067552 | -1.75 | 5.0000E-05 | 2.7364E-04 |
| 93 | CG30101 | CG30101 | 2R:13295762-13297705 | -1.75 | 5.0000E-05 | 2.7364E-04 |
| 94 | Gadd45 | Gadd45 | 2R:3136667-3138160 | -1.75 | 5.0000E-05 | 2.7364E-04 |
| 95 | CG31559 | CG31559 | 3R:1975837-1982949 | -1.75 | 5.0000E-05 | 2.7364E-04 |
| 96 | CG12918 | CG12918 | 2R:5936984-5939682 | -1.74 | 5.0000E-05 | 2.7364E-04 |
| 97 | CG33169 | CG33169 | 3L:22856963-22857596 | -1.73 | 5.0000E-05 | 2.7364E-04 |
| 98 | CG30195 | CG30195 | 2R:18534117-18534746 | -1.73 | 5.0000E-05 | 2.7364E-04 |
| 99 | CG15362 | CG15362 | 2L:1981309-1981927 | -1.71 | 5.0000E-05 | 2.7364E-04 |

| | | | | | | |
|---|---|---|---|---|---|---|
| 100 | CG9186 | CG9186 | 3L:1305274-1313377 | -1.70 | 5.0000E-05 | 2.7364E-04 |
| 101 | pip | pip | 3L:19287868-19330699 | -1.70 | 5.0000E-05 | 2.7364E-04 |
| 102 | CG12063 | CG12063 | 3R:27324960-27332716 | -1.68 | 5.0000E-05 | 2.7364E-04 |
| 103 | CG8249 | CG8249 | 2R:11731594-11734865 | -1.67 | 5.0000E-05 | 2.7364E-04 |
| 104 | CG14470 | CG14470 | 2R:1812361-1818868 | -1.67 | 5.0000E-05 | 2.7364E-04 |
| 105 | Trim9 | Trim9 | 2L:10544880-10627374 | -1.67 | 5.0000E-05 | 2.7364E-04 |
| 106 | kn | kn | 2R:10660151-10694566 | -1.65 | 5.0000E-05 | 2.7364E-04 |
| 107 | CG6287 | CG6287 | 2L:11151505-11155716 | -1.64 | 5.0000E-05 | 2.7364E-04 |
| 108 | Cpr47Ee | Cpr47Ee | 2R:7152630-7154361 | -1.64 | 4.0000E-04 | 1.7372E-03 |
| 109 | Cat | Cat | 3L:18815705-18821294 | -1.63 | 5.0000E-05 | 2.7364E-04 |
| 110 | Cpr31A | Cpr31A | 2L:10054214-10055935 | -1.62 | 5.0000E-05 | 2.7364E-04 |
| 111 | CG11380 | CG11380 | X:1085443-1090705 | -1.62 | 1.0000E-04 | 5.1606E-04 |
| 112 | dy | dy | X:11663805-11672709 | -1.61 | 5.0000E-05 | 2.7364E-04 |
| 113 | Oscillin | Oscillin | 2L:5547202-5549614 | -1.61 | 5.0000E-05 | 2.7364E-04 |
| 114 | CG11073 | CG11073 | 2R:17978993-17987231 | -1.61 | 5.0000E-05 | 2.7364E-04 |
| 115 | rdo | rdo | 2L:18012406-18070249 | -1.60 | 5.0000E-05 | 2.7364E-04 |
| 116 | CG13114 | CG13114 | 2L:9545810-9547424 | -1.59 | 1.0000E-04 | 5.1606E-04 |
| 117 | CG32572 | CG32572 | X:16608479-16620988 | -1.59 | 1.2000E-03 | 4.5893E-03 |
| 118 | Doc3 | Doc3 | 3L:8998297-9000351 | -1.59 | 5.0000E-05 | 2.7364E-04 |
| 119 | betaTub60D | betaTub60D | 2R:20193404-20200626 | -1.59 | 5.0000E-05 | 2.7364E-04 |
| 120 | CG13737 | CG13737 | 3L:13865828-13895161 | -1.58 | 5.0000E-05 | 2.7364E-04 |
| 121 | Cyp310a1 | Cyp310a1 | 2L:18617256-18661787 | -1.58 | 5.0000E-05 | 2.7364E-04 |
| 122 | SPE | SPE | 3R:19511978-19513869 | -1.57 | 5.0000E-05 | 2.7364E-04 |
| 123 | CG42324 | CG42324 | 3L:3485641-3508533 | -1.57 | 5.0000E-05 | 2.7364E-04 |
| 124 | if | if | X:16646221-16677467 | -1.57 | 5.0000E-05 | 2.7364E-04 |
| 125 | CG4914 | CG4914 | 3L:14665967-14740971 | -1.56 | 5.0000E-05 | 2.7364E-04 |
| 126 | MESR3 | MESR3 | 2L:18617256-18661787 | -1.55 | 5.0000E-05 | 2.7364E-04 |
| 127 | bip1 | bip1 | 3L:8047432-8051811 | -1.55 | 5.0000E-05 | 2.7364E-04 |
| 128 | CG31051 | CG31051 | 3R:24345269-24346237 | -1.55 | 5.0000E-05 | 2.7364E-04 |
| 129 | Epac | Epac | 2R:2649416-2684421 | -1.54 | 5.0000E-05 | 2.7364E-04 |
| 130 | Ppn | Ppn | 3R:24346386-24366053 | -1.53 | 5.0000E-05 | 2.7364E-04 |
| 131 | CG11899 | CG11899 | 3R:25026173-25028247 | -1.51 | 5.0000E-05 | 2.7364E-04 |
| 132 | snmRNA:331 | snmRNA:331 | 3R:3300564-3300674 | -1.50 | 3.8500E-03 | 1.2259E-02 |
| 133 | CG14417 | CG14417 | X:2769563-2770556 | -1.50 | 1.5350E-02 | 3.9400E-02 |
| 134 | Calx | Calx | 3R:16803997-16840182 | -1.49 | 5.0000E-05 | 2.7364E-04 |
| 135 | Nc | Nc | 3L:9961542-9964102 | -1.46 | 5.0000E-05 | 2.7364E-04 |
| 136 | CG8031 | CG8031 | 3R:8824306-8837010 | -1.46 | 5.0000E-05 | 2.7364E-04 |
| 137 | qtc | qtc | 2L:5060831-5070750 | -1.46 | 3.5000E-04 | 1.5496E-03 |
| 138 | CG8498 | CG8498 | 2L:8159839-8162389 | -1.45 | 5.0000E-05 | 2.7364E-04 |
| 139 | CG16997 | CG16997 | 2L:12111132-12111995 | -1.45 | 1.3500E-02 | 3.5442E-02 |
| 140 | Pde1c | Pde1c | 2L:11814825-11928570 | -1.45 | 5.0000E-05 | 2.7364E-04 |
| 141 | pio | pio | 2R:20468771-20485385 | -1.43 | 5.0000E-05 | 2.7364E-04 |
| 142 | CG9363 | CG9363 | 3R:5283368-5286362 | -1.43 | 1.5000E-04 | 7.3812E-04 |
| 143 | CG1273 | CG1273 | 3L:4263720-4277782 | -1.42 | 5.0000E-05 | 2.7364E-04 |
| 144 | nab | nab | 3L:4153617-4160932 | -1.42 | 5.0000E-05 | 2.7364E-04 |
| 145 | tey | tey | 3L:19643901-19656763 | -1.40 | 5.0000E-05 | 2.7364E-04 |
| 146 | CG7079 | CG7079 | 3R:16873486-16874543 | -1.39 | 2.8500E-03 | 9.5307E-03 |
| 147 | CG10932 | CG10932 | X:7781299-7782785 | -1.38 | 5.0000E-05 | 2.7364E-04 |
| 148 | daw | daw | 2L:2805517-2812147 | -1.38 | 5.0000E-05 | 2.7364E-04 |
| 149 | Sec61gamma | Sec61gamma | X:19537364-19538528 | -1.35 | 5.0000E-05 | 2.7364E-04 |
| 150 | Argk | Argk | 3L:9041961-9059127 | -1.34 | 5.0000E-05 | 2.7364E-04 |

| | | | | | | |
|---|---|---|---|---|---|---|
| 151 | Atox1 | Atox1 | 3L:21634141-21635699 | -1.33 | 5.0000E-05 | 2.7364E-04 |
| 152 | sens | sens | 3L:13389328-13394225 | -1.33 | 5.0000E-05 | 2.7364E-04 |
| 153 | Nrt | Nrt | 3L:16759173-16772844 | -1.32 | 5.0000E-05 | 2.7364E-04 |
| 154 | CG13023 | CG13023 | 3L:16917437-16920534 | -1.32 | 5.0000E-05 | 2.7364E-04 |
| 155 | Spn43Aa | Spn43Aa | 2R:3035202-3036757 | -1.31 | 5.0000E-05 | 2.7364E-04 |
| 156 | Mmp2 | Mmp2 | 2R:5498643-5571356 | -1.30591 | 5.0000E-05 | 2.7364E-04 |
