# Supplementary material for: The Drosophila Duox maturation factor is a key component of a positive feedback loop that sustains regeneration signaling
Source: PLoS Genet. 2017 Jul 28;13(7):e1006937. doi: 10.1371/journal.pgen.1006937 (PMC5550008; doi:10.1371/journal.pgen.1006937)
Supplement: S1 Text — (DOCX) [file pgen.1006937.s011.docx]

Supplementary Materials:

**qPCR:**

For quantitative PCR (qPCR), 30-40 wing imaginal discs were collected in Schneider’s medium and stored at -80^0^c. RNA was extracted using Qiagen RNeasy Mini Kit (#74104) and cDNA synthesis was performed using the Superscript III First Strand Synthesis kit (#11752-050). QPCR reactions were set up using the Power SYBR Green MasterMix (ABI) and run on the ABI Step One Plus Real Time PCR System. The experiments consisted of 3 (S7 Fig) or 4 (S3 Fig) biological replicates. For each biological replicate there were three technical replicates. Gene expression was analyzed by ΔΔC_t_ method and normalized to Gapdh2 expression. Following primers were used:

| **Gene** | **Forward Primer** | **Reverse primer** | **Source** |
| --- | --- | --- | --- |
| *mol* | TGACGGCGATTCCCATTGG | ACTCATGTCATTGGCTCCCTC | FlyPrimerBank |
| *Duox* | ATGGCTGGTACAATAACCTGGC | AACCCCATCCGAATAGGAGGG | FlyPrimerBank |
| *Nox* | TACCAGAGGGATCCTCGATAAG | GGAGTTGGTCAGATGAGAGATG |  |
| *Ets21C* | ACTGCTCCATTCCAAGGTGG | GGTCGTAGTTTCCCGCAGAA | Toggweiler et al., 2016 |
| *Gapdh2* | GTGAAGCTGATCTCTTGGTACGAC | CCGCGCCCTAATCTTTAACTTTTAC | Classen et al., 2009 |

**ROS detection through H_2_-DCFDA:**

The protocol described in Owusu-Ansah et al. was followed with slight modifications. Briefly, CM-H_2_-DCFDA (Invitrogen Molecular Probes, #C6827) was reconstituted in DMSO and then mixed with 1XPBS to make 10nM, immediately before incubation. Larvae were dissected in 1XPBS, and incubated in CM-H_2_-DCFDA solution for 5 mins on a shaker followed by three quick washes in 1XPBS. Samples were rinsed twice in 1X PBS and imaginal discs immediately dissected out to mount in Vectashield with DAPI. The samples were imaged on the confocal immediately to avoid oxidation of the dye by the environment.

**pH3 Quantification:**

pH3 positive cells in the pouch region marked by Nubbin expression were counted as previously described (Fortezza et al., 2016) with slight modifications. To count pH3 cells masks of pH3 events were generated by using ‘Threshold’ (settings: ‘Li’, ‘B&W’, ‘Dark background’, ‘Stack histogram’) and ‘Remove Outliers’ (settings: radius 2.0, threshold 50, outliers ‘Dark’) functions in ImageJ. ROI drawn on the pouch region was superimposed on the pH3 masks and cells counted using ‘Analyze particles’ (settings: size ‘0 – infinity’, circularity ‘0.0- 1.0’, show ‘outliers’) function. The total numbers of particles detected were counted as pH3 positive mitotic cells.
